# Single-cell analysis of immune recognition in chronic myeloid leukemia patients following tyrosine kinase inhibitor discontinuation

**DOI:** 10.1038/s41375-023-02074-w

**Published:** 2023-11-02

**Authors:** Jani Huuhtanen, Shady Adnan-Awad, Jason Theodoropoulos, Sofia Forstén, Rebecca Warfvinge, Olli Dufva, Jonas Bouhlal, Parashar Dhapola, Hanna Duàn, Essi Laajala, Tiina Kasanen, Jay Klievink, Mette Ilander, Taina Jaatinen, Ulla Olsson-Strömberg, Henrik Hjorth-Hansen, Andreas Burchert, Göran Karlsson, Anna Kreutzman, Harri Lähdesmäki, Satu Mustjoki

**Affiliations:** 1 Translational Immunology Research Program and Department of Clinical Chemistry and Hematology, University of Helsinki, Helsinki, Finland; 2 Hematology Research Unit Helsinki, Helsinki University Hospital Comprehensive Cancer Center, Helsinki, Finland; 3 Department of Computer Science, Aalto University, Espoo, Finland; 4 iCAN Digital Precision Cancer Medicine Flagship, Helsinki, Finland; 5 Foundation for the Finnish Cancer Institute, Helsinki, Finland; 6 Division of Molecular Hematology, Lund Stem Cell Center, Lund University, Lund, Sweden; 7 Histocompatibility Testing Laboratory, Finnish Red Cross Blood Service, Helsinki, Finland; 8 Department of Medical Sciences, Uppsala University and Hematology Section, Uppsala University Hospital, Uppsala, Sweden; 9 Department of Hematology, St. Olavs Hospital, Trondheim, Norway; 10 Department of Cancer Research and Molecular Medicine, Norwegian University of Science and Technology (NTNU), Trondheim, Norway; 11 Department of Hematology, Oncology and Immunology, Philipps University Marburg, and University Medical Center Giessen and Marburg, Marburg, Germany

**Keywords:** Tumour immunology, Chronic myeloid leukaemia

## Abstract

Immunological control of residual leukemia cells is thought to occur in patients with chronic myeloid leukemia (CML) that maintain treatment-free remission (TFR) following tyrosine kinase inhibitor (TKI) discontinuation. To study this, we analyzed 55 single-cell RNA and T cell receptor (TCR) sequenced samples (scRNA+TCRαβ-seq) from patients with CML (*n* = 13, *N* = 25), other cancers (*n* = 28), and healthy (*n* = 7). The high number and active phenotype of natural killer (NK) cells in CML separated them from healthy and other cancers. Most NK cells in CML belonged to the active CD56^dim^ cluster with high expression of *GZMA/B, PRF1, CCL3/4*, and *IFNG*, with interactions with leukemic cells via inhibitory *LGALS9*–*TIM3* and *PVR*–*TIGIT* interactions. Accordingly, upregulation of *LGALS9* was observed in CML target cells and *TIM3* in NK cells when co-cultured together. Additionally, we created a classifier to identify TCRs targeting leukemia-associated antigen PR1 and quantified anti-PR1 T cells in 90 CML and 786 healthy TCRβ-sequenced samples. Anti-PR1 T cells were more prevalent in CML, enriched in bone marrow samples, and enriched in the mature, cytotoxic CD8 + T_EMRA_ cluster, especially in a patient maintaining TFR. Our results highlight the role of NK cells and anti-PR1 T cells in anti-leukemic immune responses in CML.

## Introduction

The success of TKI therapy has enabled the achievement of TFR as the major, modern goal for patients with chronic-phase CML. However, several clinical trials have shown that only 30–40% of CML patients enrolled in such trials with stable deep molecular remission can maintain TFR after TKI therapy discontinuation [1–5], and only 20% of patients diagnosed with CML can achieve TFR during their therapy. Therefore, there is an unmet need to better identify the patients who can achieve this milestone and to increase TFR rates with additional combination regimens like with interferon-alpha (IFN-α) [6].

CML is known to be an immunologically active cancer, as even before the TKI era, IFN-α, allogeneic hematopoietic stem cell transplantation, and donor lymphocyte infusions offered opportunities to achieve remission [7–11]. As the quiescent CD34 + CD38-BCR-ABL1+ leukemic stem cells (LSCs) are known to be resistant to TKI treatment in vitro [12, 13], the immunological control or eradication of LSCs may contribute to successful TKI cessation. Recent work by us and others has demonstrated that higher proportion of cytotoxic CD56^dim^ NK cells before TKI cessation is associated with a better probability of TFR [14–18] and CD8 + T cell abundances and phenotypes have been associated with therapy responses [6, 9, 19].

**Fig. 1:**
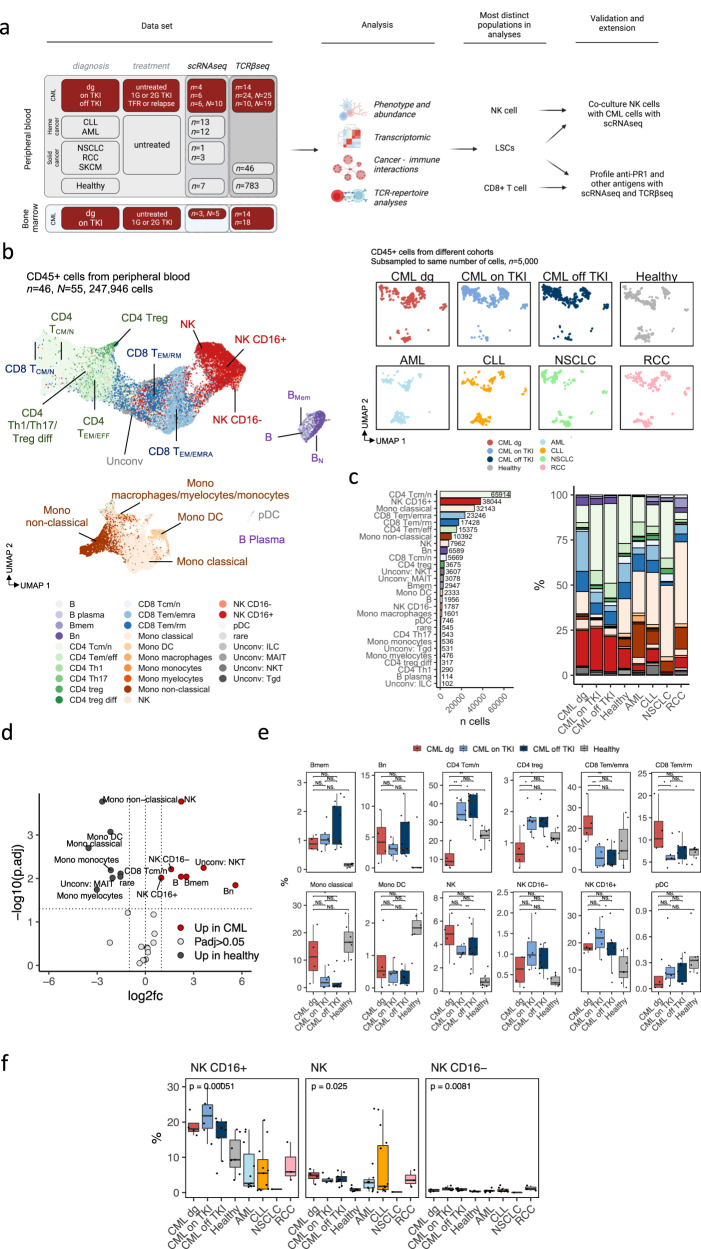
CML patients have unique NK cell repertoire. **a** Schematics showing the overview of data sets, analysis, and validation and extensions. Picture created with biorender.com. **b** Uniform Manifold Approximation and Projection (UMAP) representation of the pooled RNA profiles of 247,946 CD45+ single-cells including peripheral blood samples from patients with CML (diagnosis *n* = 4, on TKI *n* = 6, *N* = 6, off TKI *n* = 6, *N* = 10), patients with untreated hematological cancers (CLL *n* = 13, AML *n* = 11), patients with untreated solid cancer (RCC *n* = 3, NSCLC *n* = 1), and healthy controls (*n* = 7). Cells are colored based on the cluster-agnostic, reference-based method Celltypist. On the right, all different conditions are subsampled to 5,000 cells. **c** The number of predicted cell types and the median proportion of cells within each condition. Colors map to UMAP colors in **b**. **d** Differentially abundant scRNAseq populations (*P*_*adj*_ < 0.05, Benjamini-Hochberg-corrected two-sided Mann-Whitney test) between patients with CML from different disease phases (*n* = 10) and healthy controls (*n* = 7). The horizontal line denotes *P*_*adj*_ = 0.05 and the vertical lines log_2_ fold-change of 1 and −1. **e** ScRNAseq population abundances in patients with CML (diagnosis *n* = 4, on TKI *n* = 6, *N* = 6, off TKI *n* = 6, *N* = 10) and healthy controls (*n* = 7). *P*-values were calculated with a two-sided Mann-Whitney test. **f** ScRNAseq NK population abundances in patients with CML (diagnosis *n* = 4, on TKI *n* = 6, *N* = 6, off TKI *n* = 6, *N* = 10), healthy controls (*n* = 7), patients with untreated hematological cancers (CLL *n* = 13, AML *n* = 11), and patients with untreated solid cancer (RCC *n* = 3, NSCLC *n* = 1). *P* values were calculated with a Kruskal-Wallis test. CML=chronic myeloid leukemia, CLL=chronic lymphocytic leukemia, AML= acute myeloid leukemia, RCC=renal cell carcinoma, NSCLC=non-small cell lung carcinoma, TKI=tyrosine kinase inhibitor. *n* refers to the number of patients and *N* to the number of samples where it differs from *n*. *=*P* < 0.05, **=*P* < 0.01, ***=*P* < 0.001, ****=*P* < 0.0001.

To study the innate and adaptive immune responses in CML, we profiled 25 samples from CML patients including samples at diagnosis (*n* = 7, *N* = 9, where *n* is the number of subjects and *N* the number of samples if different from *n*) and before and after TKI-cessation with imatinib (*n* = 6, *N* = 16, Supplementary Fig. 1a) with paired single-cell RNA and T cell receptor (TCR) αβ chain sequencing (scRNA+TCRαβ-seq) and compared our data to different cancers (*n* = 29) and healthy donors (*n* = 7) (Supplementary Table 1) [20, 21]. Additionally, we profiled T cells specific to leukemia-associated antigen PR1 with TCRβ-sequencing (*n* = *5, N* = 12) and compared these data to unsorted TCRβ-sequencing data from CML (*n* = 35, *N* = 90) and healthy (*n* = 786) samples [22]. We validated our findings with in vitro studies including the co-culturing of immune and tumor cells (Fig. 1a).

Our high-resolution map of immune responses in CML reveals active CD56^dim^ *CD16* + *CCL4* + *IFNG* + *TIGIT* + NK cell phenotype and the inhibitory *LGALS9* – *TIM3* and *PVR* – *TIGIT* pathways controlling the NK and CML cell interactions, validated also with in vitro co-culture. Also, we provide a tool to detect anti-PR1 T cells and show their enrichment in patients with CML in comparison to healthy individuals and the bone marrow environment and how their phenotype can be associated with TFR. Our results provide insights into which aspects of anti-CML responses should be monitored before TFR or strengthened by other immunological therapies (e.g., anti-TIM3 therapies, IFN-α) in addition to TKIs to increase the rates of successful TFR.

## Results

### CML patients have NK cell biased immune repertoire in comparison to healthy and patients with other cancers

To understand the distinctive immune responses in CML, we compared CML to other hematological and solid cancers (Fig. 1a). We pooled CD45+ sorted (Supplementary Fig. 2a, b), scRNA+TCRαβ profiled blood samples from patients with CML (diagnosis *n* = 4 [6], TKI induced remission *n* = 6, TKI discontinuation *n* = 6, *N* = 10), and untreated renal cell carcinoma (RCC, *n* = 3) [20], non-small cell lung carcinoma (NSCLC, *n* = 1) [20], acute myeloid leukemia (AML, *n* = 11) [23], chronic lymphocytic leukemia (CLL, *n* = 13) [21, 24], and healthy controls (*n* = 7) [25, 26] (Fig. 1a, Supplementary Table 1).

First, we removed putative malignant cells from the data by removing a B cell cluster that contained cells only from patients with CLL and a myeloid cluster containing cells only from patients with AML (no similar cluster for patients with CML). After this, we processed 247,946 cells from these 55 CD45+ sorted peripheral blood (PB) samples with a deep generative modeling framework [27] and annotated the cell types with a cluster-agnostic approach by using an automated reference-based cell type annotation with Celltypist [28] (Fig. 1b, c). We validated the annotations with orthogonal cluster-based methods such as analyzing canonical markers, differentially expressed genes (DEGs), relationship to other clusters, signature scores, and with another automated reference-based cell type annotation SingleR [29] (Supplementary Fig. 3a–c, DEGs in Supplementary Table 2).

The most significantly expanded immune subsets in patients with CML (*n* = 10) in comparison to healthy (*n* = 7) included different NK cell populations, such as the most prevalent mature CD16 + NK cells (median out of all CD45 + 18.420% vs. 9.303%, log2 fold-change of medians [log_2_FC] = 0.986, *P*_*adj*_ = 0.00967, Benjamini-Hochberg corrected two-sided Mann-Whitney test), immature CD16- (median 0.92% vs 0.300%, log_2_FC = 1.620, *P*_*adj*_ = 0.00614), and NK cells not specified by Celltypist (median 3.625% vs 0.774%, log_2_FC = 2.228, *P*_*adj*_ = 0.00017, Fig. 1d–e). The proportion of NK cells remained increased also following TKI therapy and after TKI cessation (Fig. 1e). Also, all the NK cell phenotypes were differentially abundant across cancers (NK CD16+ *P*_*adj*_ = 0.0048; NK CD16- *P*_*adj*_ = 0.021, and NK *P*_*adj*_ = 0.048, Benjamini-Hochberg corrected Kruskal-Wallis test), and were upregulated in different phases of CML in comparison to other cancers (NK CD16+ log_2_FC = 2.157, *P*_*adj*_ = 0.00002; NK CD16- log_2_FC = 0.754, *P*_*adj*_ = 0.018; and NK log_2_FC = 0.778, *P*_*adj*_ = 0.0178, Benjamini-Hochberg corrected Mann-Whitney test, Fig. 1f, Supplementary Fig. 4a).

### T cell compartment is altered from CD8 + T_EM/EMRA_ to CD4 + T_CM/N_ and T_REG_ biased after imatinib start and remains similar following TKI cessation

The other cell types that were expanded in patients with CML in comparison to healthy included different B cells (memory B cells median 0.968% vs 0.165%, log_2_FC = 2.553, *P*_*adj*_ = 0.0091; naïve B cells 3.157% vs 0.065%, log_2_FC = 5.602, *P*_*adj*_ = 0.0143; and B cells unspecified by Celltypist 0.383% vs 0.083%, log_2_FC = 2.206, *P*_*adj*_ = 0.0091), but their levels remained quite small throughout treatment (Fig. 1d, e, Supplementary Fig. 4b).

One of the interestingly behaving cell type was the largest CD8 + T cell population CD8 + T_EM/EMRA_, whose levels were higher in CML diagnosis compared to healthy controls and other cancers (CD8 + T_EM/EMRA_ median 20.157% vs 5.495%, log_2_FC = 1.875, *P* = 0.0082, *P*_*adj*_ = 0.0662), but their levels decreased following TKI initiation (CD8 + T_EM/EMRA_ median 20.157% vs 5.623%, log_2_FC = 1.842, *P* = 0.009, *P*_*adj*_ = 0.133, Fig. 1d, e, Supplementary Fig. 4b). The inverse was seen with CD4+ regulatory T cells (T_reg_, 0.635% vs 1.404%, log_2_FC = −1.145, *P* = 0.038, *P*_*adj*_ = 0.178) and CD4+ central memory / naïve T cells (T_CM/N_, 8.804% vs 17.510%, log_2_FC = −0.992, *P* = 0.0095, *P*_*adj*_ = 0.134) whose levels rose following TKI initiation (T_reg_ 0.635% vs 1.698%, log_2_FC = −1.419, *P* = 0.038, *P*_*adj*_ = 0.178, T_CM/N_ 8.804% vs 34.161%, log_2_FC = −1.956, *P* = 0.009, *P*_*adj*_ = 0.133, Fig. 1d, e, Supplementary Fig. 4b).

Both classical (median 1.428% vs 16.536%, log_2_FC = −3.534, *P*_*adj*_ = 0.002) and non-classical monocytes (median 0.562% vs 3.615%, log_2_FC = −2.685, *P*_*adj*_ = 0.00017) were decreased in CML patients compared to healthy controls as well as dendritic cells (DCs, 0.410% vs 1.849%, log_2_FC = −2.173, *P*_*adj*_ = 0.00085, Fig. 1d, e, Supplementary Fig. 4b).

The findings made with the automated cell type predictions, including expansion of NK cells, CD8 + T cells, B cells, and depletion of monocytes in patients with CML, were validated with cluster-based analysis (Supplementary Fig. 5a, b).

### CML patients’ NK cell repertoire is enriched to active NK cell phenotype rarely seen in healthy controls

**Fig. 2:**
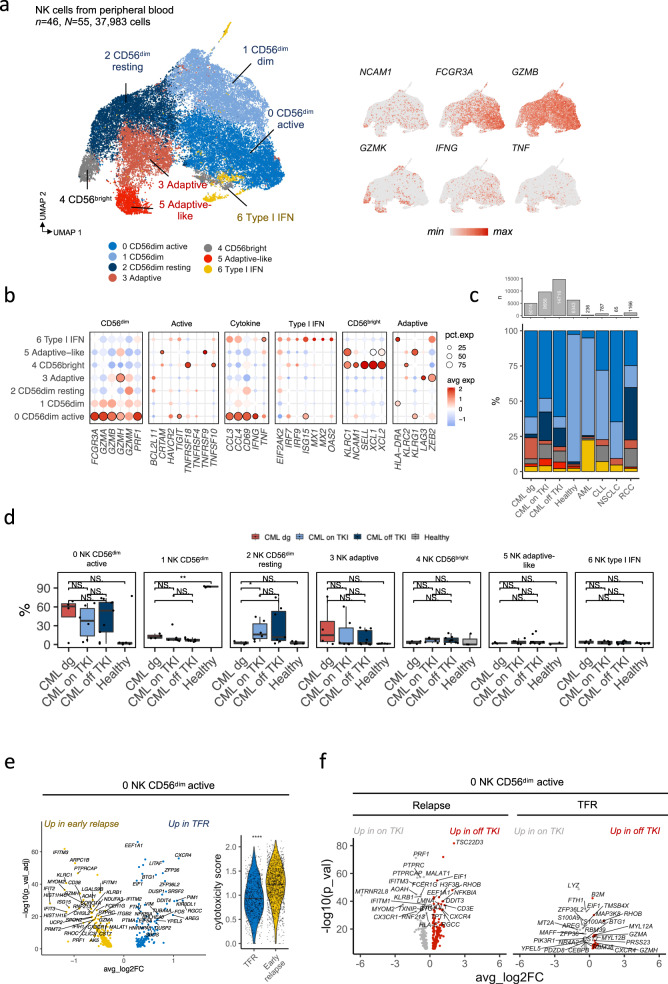
NK cell phenotype changes to more mature during the TKI treatment. **a** UMAP representation of the NK CD16 + , NK CD16-, and NK cells identified by Celltypist, colored by manually annotated clusters or scaled expression of genes used to annotate the phenotypes. **b** Scaled average expressions (avg exp) and proportion of cells expressing (pct.exp) the canonical markers used to define the clusters. Encircled dots are differentially expressed (*P*_*adj*_ < 0.05, Bonferroni corrected *t*-test) in a given cluster in comparison to other clusters. **c** Top: The number of NK cells from each condition. Bottom: The median proportion of NK cell subtypes out of total NK cells within each condition. Colors in the lower panel map to UMAP colors in **a**. **d** ScRNAseq population abundances in patients with CML (diagnosis *n* = 4, on TKI *n* = 6, *N* = 6, off TKI *n* = 6, *N* = 10) and healthy controls (*n* = 7). *P*-values were calculated with a two-sided Mann-Whitney test. **e** Left: Differentially expressed genes (*P*_*adj*_ < 0.05, Bonferroni corrected *t*-test) in active CD56^dim^ NK cells (cluster 0) from before TKI cessation between patients who sustain treatment-free remission (TFR) and patients who had early relapse ( < 6 months) after cessation. Right: Cytotoxicity score of active CD56^dim^ NK cells (cluster 0) in baseline in patients who sustain TFR and early relapse. Note that multiple genes upregulated in patients with early relapse were associated with cytotoxicity. **f** Left: Differentially expressed genes (*P*_*adj*_ < 0.05, Bonferroni corrected *t*-test) in active CD56^dim^ NK cells (cluster 0) from after and before TKI cessation, separately in TFR and early relapse. CML=chronic myeloid leukemia, CLL = chronic lymphocytic leukemia, AML= acute myeloid leukemia, RCC = renal cell carcinoma, NSCLC = non-small cell lung carcinoma, TKI = tyrosine kinase inhibitor. *N* refers to the number of patients and *N* to the number of samples where it differs from *n*. *=*P* < 0.05, **=*P* < 0.01, ***=*P* < 0.001, ****=*P* < 0.0001.

As the automated cell type algorithms are designed to capture coarse cell types and thus are not able to appreciate subtle differences seen in previous NK cell-related scRNAseq publications, we selected the NK cells and performed clustering analysis with manual annotation. From 37,983 cells, we identified 7 types of different NK cells, which is on par with the previous publications [30–33] and includes all different maturation stages of NK cells (CD56^bright^ NK → CD56^dim^ NK → adaptive NK, Fig. 2a, b, Supplementary Fig. 6a). The CD16 + NK cells predicted by Celltypist were CD56^dim^, CD16- NK cells were CD56^bright^ cells and the unassigned NK cells both CD56^bright^ and adaptive NK cells (Supplementary Fig. 6a).

The largest phenotype, CD56^dim^ cells with cytotoxic potential, formed three different clusters (clusters 0, 1, and 2), which were named following their level of presumed activation by expression of genes related to cytotoxicity (e.g., *FCGR3A/CD16*, *GZMA/B/M/H*, *PRF1*), activation (e.g., *TNFRSF4/9/10/18*, *TIGIT*, *HAVCR2/TIM3*), and cytokine secretion (e.g., *CCL3/4, TNF, IFNG*). Interestingly, the cluster deemed most active (cluster 0) was enriched in patients with CML and only seen in small quantities in healthy (median % out of all NK cells 54.100% vs 2.610%, log_2_FC = 4.373, *P* = 0.015, two-sided Mann-Whitney test, Fig. 2c, d).

Other NK cell clusters, like CD56^bright^ cells (cluster 4) were defined by expression of *CD56* (*NCAM1*), *SELL, XCL1/2*, and *GZMK* as previously [30–33] and formed the 5^th^ smallest population, whose levels arose following TKI therapy, albeit finding was statistically insignificant. Adaptive and adaptive-like NK cells (cluster 3 and 5), which were defined by expression of *ZEB2*, *KLRC2*, *GZMH*, and lack of TCRs as in the previous publications [30–33], were enriched in cytomegalovirus (CMV) seropositive donors (cluster 3, cells from CMV + 90.51% and CMV- 9.49%, *P* < 2.2 × 10^−16^; and cluster 5 cells from CMV + 88.70% and CMV- 11.30%, *P* < 2.2 × 10^−16^, two-sided Fisher’s exact tes**t**, Supplementary Fig. 6b). In CMV+ patients (*n* = 3), the adaptive NK cell cluster (cluster 3), was abundant during diagnosis (median 5.68%), rose on-TKI (median 15.40%), and decreased to diagnosis levels off-TKI (median 6.33%).

### NK cells have an active phenotype in long-term TFR

During imatinib treatment (on TKI samples), the level of resting NK cells (cluster 2) increased compared to the time of diagnosis (cluster 2, median 1.86% vs 15.80%, log_2_FC = 3.088, *P* = 0.0095, Fig. 2d). However, after imatinib cessation, the levels of active NK cells (cluster 0) increased (37.10% vs 51.80%, log_2_FC = 0.482) resting NK cells decreased (median off-TKI 11.30%, log_2_FC = 0.484), but differences were not statistically significant (*P* > 0.05). Interestingly, the levels of active NK cells (cluster 0) seemed to be similarly high at diagnosis (median 37.10%), at relapse following TKI cessation (median 69.60%), and at 1 year in TFR (median 59.10%, Supplementary Fig. 6c).

At the time of TKI cessation, the active NK cells (cluster 0) had upregulated expression of cytotoxicity-related genes (*GMZA/B/H, PRF1*), translating into a higher cytotoxicity score in patients who relapsed compared to patients maintaining TFR (median score 0.935 vs 0.724, log_2_FC = 0.369, *P* < 2.2 × 10^−16^, Fig. 2e). When comparing pre- and post TKI cessation samples, patients with TFR had a lower number of DEGs than patients with relapse (553 vs 64). In post-TKI cessation samples from patients sustaining TFR, CD56^dim^ active NK cells had increased expression of *GZMA/H*, *CXCR4*, and *IFNG* compared to pre-TKI cessation samples, and this was not observed in patients who relapsed (Fig. 2f, DEGs in Supplementary Table 2).

### Immune activity is higher towards cells with higher BCR-ABL1 pathway activity

**Fig. 3:**
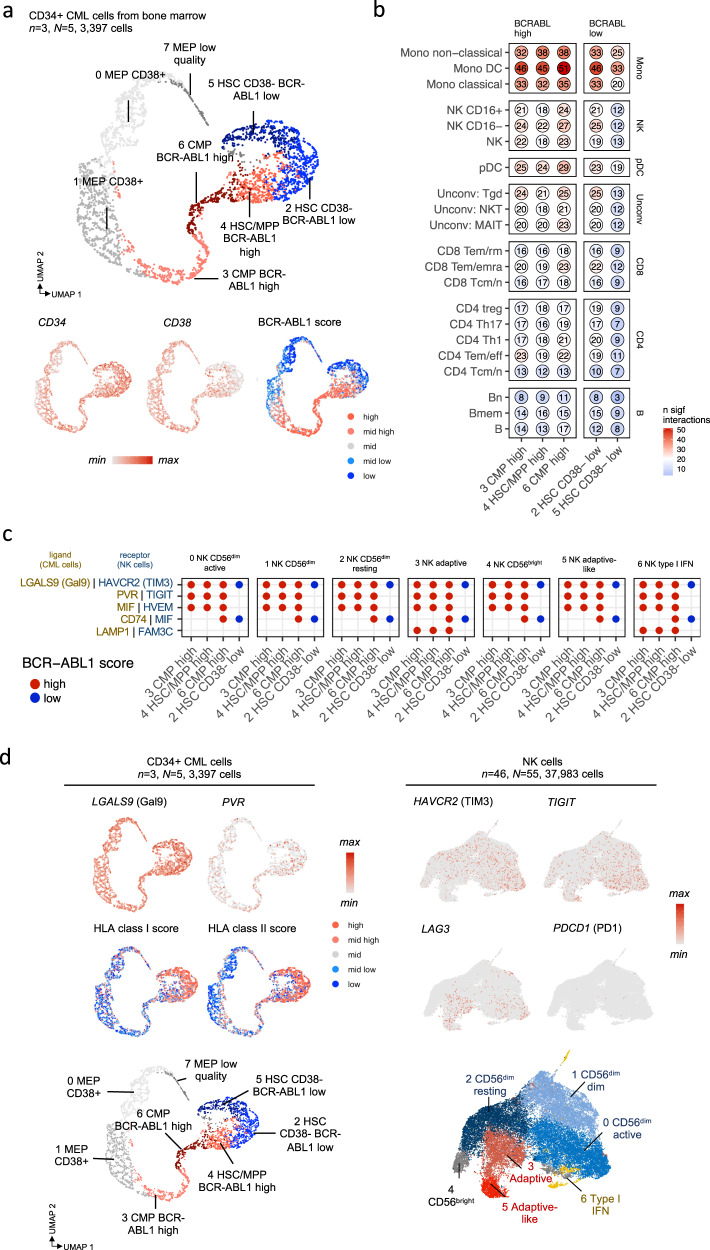
Immune interactions are more frequent against CD34+ cells that express high BCR-ABL1 pathway activity. **a** UMAP representation of the pooled RNA profiles of patients with newly diagnosed CML (*n* = 3) CD34+ (*N* = 3) and CD34 + CD38- (*N* = 2) sorted single-cells from the bone-marrow, colored by manually annotated clusters or scaled expression of genes used to annotate the phenotypes or BCR-ABL1 activity score shown in five equal quantiles. BCR-ABL1 score was calculated with differentially expressed genes between BCR-ABL1 mutated and wild-type CML cells in Giustacchini et al. [37]. **b** The number of significant (*P* < 0.05, CellPhoneDB test) ligand-receptor interactions between CML cells and immune cells from patients with newly diagnosed CML. **c** Significant (*P* < 0.05, CellPhoneDB test) inhibitory ligand-receptor interactions between CML and NK cells. Ligands that are expressed on CML cells are highlighted in brown color and receptors that are expressed on immune cells are highlighted in blue color. Interactions are shown separately with different CML CD34+ cell clusters, where CML CD34+ clusters with high BCR-ABL1 score are highlighted in red and CD34+ with low BCR-ABL1 score are highlighted in blue. **d** Expression of inhibitory ligand-receptor genes and signature scores in CML LSCs (the same UMAP representation as in Fig. 3a) and NK cells (the same UMAP representation as in Fig. 2a). *n* refers to the number of patients and *N* to the number of samples where it differs from *n*.

It is essential to understand how leukemic CML cells are recognized or evaded from the immune system. To study the interactions between CML and immune cells, we profiled CD34+ and CD34 + CD38- populations from 3 additional diagnostic phase CML patients’ bone marrow samples. We received 8 different clusters, annotated manually with canonical markers [34, 35] and Nabo [36], including primitive CD34 + CD38- hematopoietic stem cells (HSCs, clusters 2, 4, and 5), more mature common myeloid progenitors (CMPs, clusters 3 and 6), and megakaryocytic-erythroid progenitors (MEP, clusters 0, 1, and 7, Fig. 3a, Supplementary Fig. 7a–c, DEGs in Supplementary Table 2). In the CD34 + CD38- sorted samples, the percentage of cells belonging to clusters named as HSCs (clusters 2, 5) was higher than in CD34+ sorted samples (median of HSC named cells of all cells 93.4% vs 33.7%, log_2_FC = 1.47, Supplementary Fig. 7b).

We analyzed the BCR-ABL1 pathway activity of these clusters with DEGs between BCR-ABL1+ and BCR-ABL1- HSCs from a previous scRNAseq study [37] (genes described in Methods). Three out of eight clusters had higher BCR-ABL1 -scores than the other clusters (median scores 0.377 vs 0.297, log_2_FC = 0.344, *P* < 2.2 × 10^−16^, two-sided Mann-Whitney), including one HSC/MPP (cluster 4, median score 0.384) and two CMP-clusters (cluster 3 and 6, median scores 0.424 and 0.418), suggesting that the most primitive HSC clusters are not highly dependent on BCR-ABL1 activity in accordance with previous findings [13] (Fig. 3a).

We predicted the immune cell interactions between the BM CML cells and the PB immune cells from other newly diagnosed CML by calculating significant (*P* < 0.05, CellPhoneDB permutation test) ligand-receptor interactions with CellPhoneDB [38] (Supplementary Table 3). Interestingly, the immune system had more predicted interactions with populations that had higher BCR-ABL1-pathway activity (median interactions 17 vs 12.5, log_2_FC = 0.444, *P* = 3.8 × 10^−5,^, Fig. 3b, Supplementary Fig. 8a). The immune cells that interacted the most with CML cells included different myeloid cells (DCs 211 interactions, non-classical 150, and classical monocytes 133), and different NK cell populations (NK CD16- 96, NK CD16 + 79, NK 76, Fig. 3b).

### NK cells interact with CML cells via the inhibitory LGALS9 - HAVCR2 and PVR – TIGIT axes

Albeit the myeloid cells had the most predicted interactions with the tumor cells, we wanted to focus on NK cells as they had come up as top hits in our analyses of cell abundance and transcriptomic profiles. The ligand-receptor interactions between CD34 + LSC cells and different NK cell subpopulations could be divided into different categories, including HLA interactions (e.g., *HLA-E* and *KLRC1/2*), cell adhesion molecules (e.g., *SELL/SELP* and *SEPLG*), TRAIL (e.g., *TRAIL* and *RIPK1*), and co-stimulatory interactions (e.g., *CLEC2B* and *NKp80*, Supplementary Fig. 8b). The ligand-receptor interactions were quite uniform across different CD34 + CML cells and NK cell subpopulations, although interactions against CD34 + CML with low BCR-ABL1 activity were scarce.

Importantly, one of the major interaction categories included inhibitory interactions, where the most abundant involved *LGALS9* encoding Galectin-9 and *HAVCR2/TIM3* (Fig. 3c). *LGALS9* was expressed by CD34+ LSCs with low and high BCR-ABL1 activity, but the highest expression was found in the most primitive CD34 + CD38- cells (cluster 2, 4 and 5), where also the inhibitory HLA class I and II expressions were found (Fig. 3d). The highest *HAVCR2/TIM3* expression was identified in the CD56^dim^ active cluster (cluster 0, Fig. 3d). Although *PVR* was expressed by lower levels of CML cells than *LGALS9* and *TIGIT* lower than *HAVCR2/TIM3* on NK cells, the interactions involving *PVR* and *TIGIT* were abundant. Similarly to *LGALS9*, *PVR* was expressed by CD34+ LSCs with low and high BCR-ABL1 activity with the highest in BCR-ABL1 + GMP cells (cluster 3 and 6), and the highest *TIGIT* was expressed by active NK cells, including CD56^dim^ active (cluster 0) followed by type I IFN NK cells (cluster 6, Fig. 3c, d, Supplementary Fig. 8c).

### NK cells transfer to an active phenotype when co-cultured with CML cells

We next sought to validate and refine the identified NK cell populations and interactions between CML and NK cells in an experimental co-culture model. We previously examined the transcriptomic profiles of interacting NK cells and a panel of blood cancer cell lines [33]. To focus particularly on CML, we co-cultured two different CML cell lines (K562 and LAMA84) with and without primary NK cells that were either unexpanded or expanded with IL-2 and feeder cells and profiled these 6 different conditions with scRNAseq as previously described [33].

**Fig. 4:**
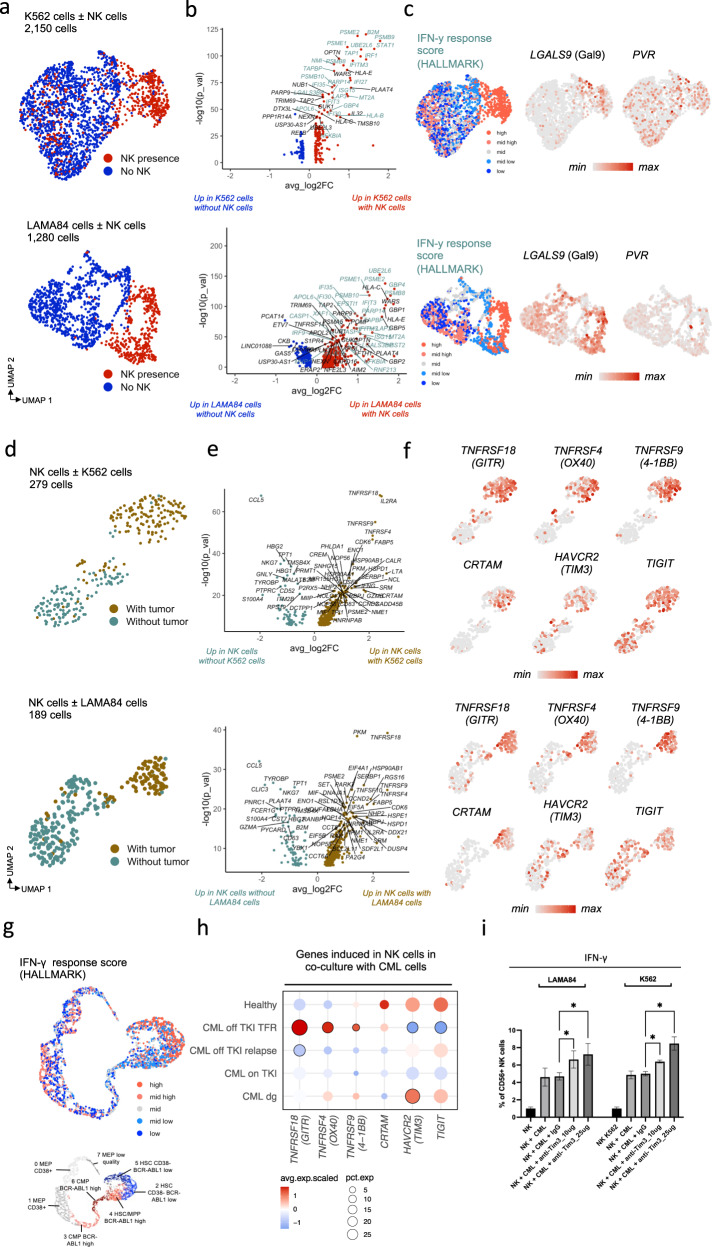
Co-culture of expanded NK cells with CML cell lines show upregulated IFN-γ response in CML cells and active phenotype of NK cells. **a** UMAP representation of K562 and LAMA84 cells cultured with and without expanded NK cells, colored by the presence of expanded NK cells. **b** Differentially expressed genes (*P*_*adj*_ < 0.05, Bonferroni corrected *t*-test) between K562 and LAMA84 cells cultured with and without expanded NK cells. The genes related to IFN-γ response HALLMARK-category are highlighted in teal color. **c** Expression of selected co-inhibitory genes and IFN-γ response score in K562 and LAMA84 cells, calculated with the HALLMARK-category genes and shown in five equal quantiles. **d** UMAP representation of expanded NK cells cultured with and without K562 or LAMA84 cells, colored by the presence of tumor cells. **e** Differentially expressed genes (*P*_*adj*_ < 0.05, Bonferroni corrected *t*-test) between expanded NK cells cultured with and without K562 or LAMA84 cells. **f** Expression of selected co-stimulatory genes in NK cells with and without co-culture of CML cells. **g** Expression of IFN-γ response score in primary CML LSCs. **h** Scaled average expressions (avg exp) and proportion of active NK cells expressing (pct.exp) some genes induced in the co-culture of NK cells, in patient samples. Encircled dots are differentially expressed (*P*_*adj*_ < 0.05, Bonferroni corrected *t*-test) in a given patient group in comparison to other groups. **i** Proportion of expanded NK cells expressing IFN-γ after co-culture with CML cell lines LAMA84 or K562 with or without TIM3 blocking antibody in different concentrations. *P*-values were calculated with a paired *t*-test. *=*P* < 0.05, **=*P* < 0.01, ***=*P* < 0.001, ****=*P* < 0.0001.

Most of the variance between the CML cells was explained by the co-culture with NK cells (Fig. 4a). CML cell lines experienced a strong IFN-γ response following the NK cell attack. The top significant DE-genes (*P*_*adj*_ < 0.05, Bonferroni corrected t-test) included class I HLA genes (*B2M*, *HLA*-*A/B/C/E*), class II HLA genes (*HLA-DRA, HLA-DRB1, HLA-DMA*), antigen processing machinery (*TAP1, TAP2*), immunoproteasome genes (*PSMB8/9/10), PSME1/2*), and JAK-STAT genes (*STAT1, IRF1*, Fig. 4b, Supplementary Table 2). The co-culture of NK cells with CML cells induced strong upregulation of *LGALS9* on CML cells, and the top hits in the interaction analysis included *LGALS9* – *TIM3* and *PVR* – *TIGIT* (Fig. 4c). *PVR* was not similarly upregulated on CML cells as *LGALS9* upon co-culture with NK cells. The findings were validated with non-expanded primary NK cells, where the effects of NK cells were even more pronounced in LAMA84 than in K562 cells (Supplementary Fig. 9a–c).

Accordingly, NK cells gained an active phenotype when cocultured with CML cells (Fig. 4d). The top significant DE-genes (*P*_*adj*_ < 0.05) between NK cells cultured alone and in co-culture with CML cells were different costimulatory genes such as *TNFRSF9* (*4-1BB*), *TNFRSF18* (*GITR*), *TNFRSF4* (*OX-40*), and *CRTAM*, but also cytotoxicity genes like *IFNG* and *GZMB*, as also previously observed for K562 and certain other cell lines [33]. The downregulated genes included other cytotoxicity genes such as *GNLY*, *CCL5*, *NKG7*, and *GZMA* (Fig. 4e, Supplementary Table 2). The expression of *LGALS9* receptor *HAVCR2* (*TIM-3*) and *PVR* receptor *TIGIT* were not statistically significantly altered in NK cells, possibly due to high baseline expression (Fig. 4f). The findings were validated with the non-expanded primary NK cells, and the active phenotype of NK cells was seen in both LAMA84 and K562 co-cultures, but even stronger when NK cells were co-cultured with LAMA84 cells (Supplementary Fig. 9d–f).

In the CML LSCs from patients, the IFN-γ response signature was the highest in the most primitive CD34 + CD38- cells with low BCR-ABL1 activity (clusters 2, and 5) (Fig. 4g). The genes induced in NK cells in co-culture with CML cells that were attributed with co-stimulatory functions like *TNFRSF9*/*4-1BB* (*P*_*adj*_ = 0.0026, Bonferroni corrected t-test), *TNFRSF18*/*GITR* (*P*_*adj*_ = 6.05 × 10^−11^), and *TNFRSF4*/*OX-40* (*P*_*adj*_ = 0.0038) were significantly upregulated in active NK cells in patients with TFR in comparison to on TKI and relapse samples (all *P*_*adj*_ < 0.01, Bonferroni corrected t-test). Further, the inhibitory genes *HAVCR2/TIM3* (*P*_*adj*_ = 6.63 × 10^−5^) and *TIGIT* (*P*_*adj*_ = 0.00043) were downregulated in patients maintaining TFR in comparison to relapse samples (Fig. 4h, Supplementary Table 2).

### Blocking TIM3 enhances NK cell IFN-γ secretion

To functionally test the importance of LGALS9 and TIM3 interaction in the NK cell activation, we blocked TIM3 with a clinical grade antibody sabatolimab [39] in varying concentrations (1, 10, 25 μg/ml) with expanded NK cells from two healthy donors co-cultured with K562 and LAMA84 cell lines. The impact on the NK cell phenotype was analyzed by measuring degranulation (CD107a) and cytokine secretion (IFN-γ and TNF-α) and the cell killing with a luciferase-based assay.

After blocking TIM3, we noted a dose-dependent increase in IFN-γ secretion in expanded NK cells against K562 (IgG vs 10 μg/ml vs 25 μg/ml, mean % of NK cells 5.06% vs 6.39% vs 8.47%, IgG vs 10 μg/ml *P* = 0.028 and IgG vs 25 μg/ml *P* = 0.021, paired *t*-test) and LAMA84 (IgG vs 10 μg vs 25 μg, mean % of NK cells 4.69% vs 6.63% vs 7.22%, IgG vs 10 μg/ml *P* = 0.042 and IgG vs 25 μg/ml *P* = 0.049, Fig. 4i). The degranulation (CD107a) and TNF-α secretion remained similar (Supplementary Fig. 10a), in keeping with previous publications with primary CML samples [40]. The findings were replicated in another NK cell donor (Supplementary Fig. 10b). In the luciferase-based assay, we did not notice any enhanced killing by expanded NK cells by blocking TIM3 in varying concentrations against K562 or LAMA84 (Supplementary Fig. 10c).

We also tested primary sorted CD34+ cells from one CML patient in a co-culturee with non-autologous expanded NK cells from a healthy donor with different TIM3 antibody concentrations (1 and 10 μg/ml). Although expanded NK cells were able to kill CD34 + CML cells, no enhanced cell killing was noted with TIM3 blockade in CD34+ cells or leukemic stem cells CD34 + CD38- cells (Supplementary Fig. 10d). Also, the degranulation, IFN-γ secretion, and TNF-α secretion levels of expanded NK cells remained similar (Supplementary Fig. 10e).

### TCRs from anti-PR1 T cells show common patterns that are exploitable by machine learning

**Fig. 5:**
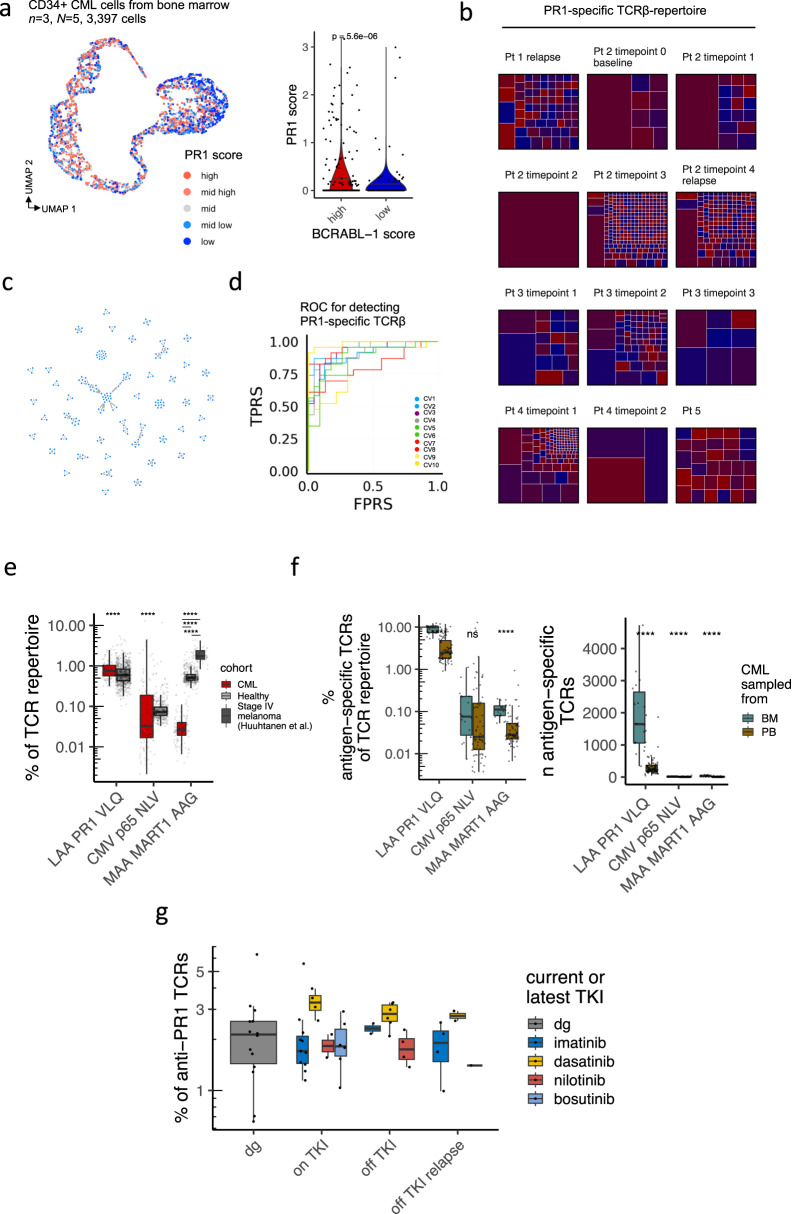
Monitoring antigen-specific T-cell responses in CML show expansion of anti-PR1 T cells in CML, in bone marrow, and in dasatinib-treated patients. **a** The same UMAP representation as in Fig. 3A of the pooled RNA profiles of patients with newly diagnosed CML (*n* = 3) CD34+ (*N* = 3) and CD34 + CD38- (*N* = 2) sorted single-cells from the bone-marrow, colored by PR1-expression score, which is a combination of *PRTN3* and *ELANE* genes carrying the PR1 epitope. Clusters were divided into BCR-ABL1 high and low based on the clusters in Fig. 3A. *P* value was calculated with a two-sided Mann-Whitney. **b** Clonal structure of anti-PR1 T cell clonotype in individual samples. Each box within a facet is a unique clonotype, where its size corresponds to its proportion in the TCR repertoire. The same clonotypes are colored with the same color. **c** Network graph showing the similarity of the 231 anti-PR1 specific TCRs. Each node is a unique TCR and an edge between nodes denotes amino-acid-level similarity determined by GLIPH2. **d** AUROC plot showing the 10-fold cross-validation of the 231 anti-PR1 TCRs used as input for the TCRGP-classifier, where TPRS denotes true positive rates and FPRS denotes false positive rates. The mean of the AUROCs was 0.902. **e** The proportion of TCRGP predicted antigen-specific TCRs in CML (*n* = 48), healthy donors’ (*n* = 786), and in patients with melanoma (*n* = 46) peripheral blood samples. The samples were subsampled to the same sequencing depth. *P*-value was calculated with a two-sided Mann-Whitney test. **f** The proportion and number of TCRGP predicted antigen-specific TCRs in patients with CML in bone marrow (BM, *n* = 15) and peripheral blood (PB, *n* = 24). The samples were subsampled to the same sequencing depth. *P*-values were calculated with a two-sided Mann-Whitney test. **g**) The proportion of anti-PR1 T cells in patients with CML in diagnosis (*n* = 14), on TKI (*n* = 23), off TKI (*n* = 12), off TKI relapse (*n* = 7). *P*-values were calculated with a two-sided Mann-Whitney test. *n* refers to the number of patients and *N* to the number of samples where it differs from *n*. *=*P* < 0.05, **=*P* < 0.01, ***=*P* < 0.001, ****=*P* < 0.0001.

Antigen-specific T cells are fundamental for understanding and monitoring cancer immunity. To study antigen-specific T cells in CML, we chose PR1, a leukemia-associated antigen from proteinase 3 (*PRTN3*) and neutrophil elastase (*ELANE*) genes, as anti-PR1 T cells have been associated with response to TKI [7] and successful IFN-α discontinuation [41]. In our scRNAseq data of CD34 + CML cells, both *PRTN3* and *ELANE* were significantly upregulated in CML cells with high BCR-ABL1 score (median pooled PR1 score 0.25 vs 0.11, log_2_FC = 1.184, *P* = 5.6 × 10^−6^, two-sided Mann-Whitney test, Fig. 5a).

We sorted PR1 specific CD8 + T cells from 12 samples from 6 CML patients with HLA-A*02:01 PR1-targeting peptide-major histocompatibility complex (pMHC) tetramers, analyzed them with TCRβ-sequencing and received 772 PR1-specific TCRβs (Fig. 5b). Even though the PR1-epitope is the same in all individuals, we found that the TCRs recognizing it differ largely. Approximately only one-third of the PR1-specific TCRs had exact matches in a previously published cohort of 786 healthy donors’ TCR repertoire [22] (Supplementary Fig. 11a).

As 2/3 of the PR1-specific sequences were private to individuals, it hinders the direct identification of anti-PR1 clonotypes from samples where no anti-PR1 enrichment is done. However, we reasoned that at least a subgroup of PR1-specific TCRs could share amino acid level similarities across patients and thus grouped TCRs with an unsupervised algorithm GLIPH2 [42]. We found that 231/772 of anti-PR1 TCRs shared peptide motifs, showing that there are common patterns of how PR1 is recognized by the T cells across patients (Fig. 5c, full GLIPH2 results in Supplementary Table 4).

Albeit GLIPH2 was useful as it identified anti-PR1 TCR motifs, GLIPH2 cannot be used as a predictive model per se. Therefore, we used our recently described method TCRGP [43] to train a machine learning classifier to detect PR1-specific TCRs from previously unseen data, where no PR1-sorting has been done. We used the identified 231 PR1-specific TCRs and 462 non-specific TCRs as controls to train a PR1-specific classifier with TCRGP, a framework that is used widely in TCR-prediction tasks [44–47]. In 10-fold cross-validation, we received a mean area under the curve (AUROC) of 0.902 for detecting anti-PR1 TCR sequences, providing us with a tool to identify anti-PR1 specific TCRs (Fig. 5d). Our TCRGP model uses only the CDR3β, thus focusing on the epitope rather than the HLA-molecule which is within more close proximity of the Vβ and Jβ parts of the TCR [48].

### Anti-PR1 T-cells are found more commonly in CML than in healthy, expanded in bone marrow, and elevated by dasatinib treatment

We predicted PR1-specific TCRs with our TCRGP classifier from 90 TCRβ-sequenced CML samples from 35 patients and 786 healthy samples. To compare, we also predicted antigen-specific TCRs against common viruses (CMV, EBV, Influenza A, and HSV-2) and a cancer-associated antigen unrelated to CML, namely MART1 [44] (TCRGP results in Supplementary Table 4). It should be noted that these samples were not HLA-genotyped due to sample scarcity, but we expect balanced ratio of HLA-A*02:01 positive samples across groups.

In comparison to healthy, CML patients had significantly expanded anti-PR1 responses (median % of total TCR repertoire 0.743% vs 0.595%, log_2_FC = 0.320, *P* = 4.9 × 10^−5^, two-sided Mann-Whitney test, Fig. 5e). In comparison to the anti-viral antigens and the melanoma-associated antigens, the anti-PR1 was the most expanded antigen-specific response in CML patients (0.743% vs 0.0226%, log_2_FC = 5.039, *P* = 2.2 × 10^−16^, Fig. 5e, Supplementary Fig. 11b). In patients with CML, the bone marrow had a higher number of anti-PR1 TCR clones than peripheral blood (median 686 vs 92, log_2_FC = 2.899, *P* = 8.9 × 10^−10^), and these also occupied the larger part of the repertoire (median % of total TCR repertoire 4.33% vs 0.743%, log_2_FC = 2.543, *P* = 1.7 × 10^−8^, Fig. 5f, Supplementary Fig. 11c).

The frequency of anti-PR1 was related to different therapies in patients with CML. Patients that received dasatinib had higher levels of anti-PR1 T cells than patients receiving imatinib or other 2^nd^ generation TKIs (3.30% vs 1.79%, log_2_FC = 0.882, *P* = 0.0061, two-sided Mann-Whitney test, Fig. 5g), even though their clonalities were not significantly different (*P* = 0.12, Supplementary Fig. 11d). The levels of anti-PR1 T cells remained similar when TKI therapy was discontinued, and patients with prior dasatinib treatment had higher levels of anti-PR1 T cells than patients that had other TKIs prior discontinuation (2.82% vs 2.05%, log_2_FC = 0.460, *P* = 0.015, Fig. 5g).

### Anti-PR1 response is more exhaustive and less cytotoxic in early relapse than in TFR patient

To understand phenotypic differences of PR1-specific T cells in individual patients in a TKI discontinuation setting, we utilized paired scRNA+TCRαβ-seq and PR1-specific TCRβ-seq data from 2 patients, and thus were able to hard-match the PR1-specific clonotypes in these samples. On-TKI, the anti-PR1 enriched TCRs explained 3.9% of the repertoire in the TFR patient (Patient 5) and 3.2% in the early relapse patient profiled with scRNA+TCRαβ-seq (Patient 7), which were comparable to the TCRGP predicted levels in the TCRβ-seq samples (Supplementary Fig. 12a, b). Although after TKI withdrawal, the abundance of anti-PR1 clones remained similar in the different outcomes, 4/5 of the largest clones involuted over twofold in the relapsing patient compared to 1/5 in the TFR patient following TKI cessation attempt (Supplementary Fig. 12c).

**Fig. 6:**
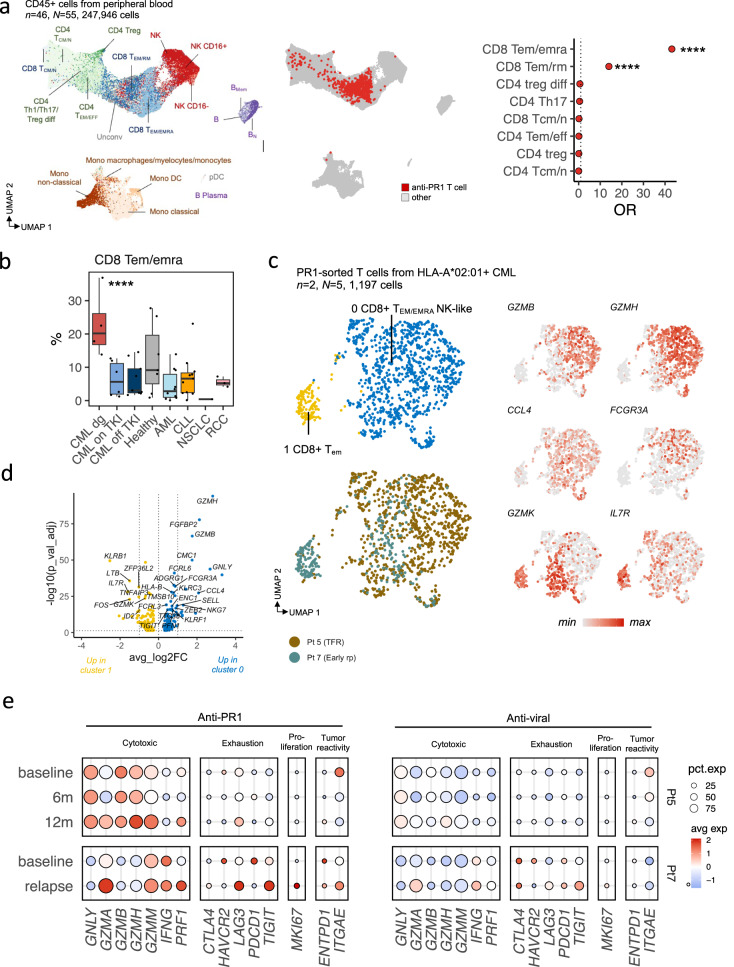
Phenotype of antigen-specific T cells show upregulated cytotoxicity and lesser exhaustion in anti-PR1 T cells in TFR in comparison to early relapse. **a** The same UMAP representation as in Fig. 1A of the pooled RNA profiles of 247,946 CD45+ single-cells including peripheral blood samples from patients with CML (diagnosis *n* = 4, on TKI *n* = 6, *N* = 6, off TKI *n* = 6, *N* = 10), patients with untreated hematological cancers (CLL *n* = 13, AML *n* = 11), patients with untreated solid cancer (RCC *n* = 3, NSCLC *n* = 1), and healthy controls (*n* = 7), where T cells specific to PR1 are highlighted. The odds ratios (OR) of anti-PR1 T cells present in distinct clusters were calculated with the cell types shown in Fig. 1A. **b** ScRNAseq NK population abundances in patients with CML (diagnosis *n* = 4, on TKI *n* = 6, *N* = 6, off TKI *n* = 6, *N* = 10), healthy controls (*n* = 7), patients with untreated hematological cancers (CLL *n* = 13, AML *n* = 11), and patients with untreated solid cancer (RCC *n* = 3, NSCLC *n* = 1). *P*-value was calculated with a Kruskal-Wallis test. **c** UMAP representation of T cells with known anti-PR1 specificity from a patient with a treatment-free remission (*N* = 3 samples from 3 timepoints) and a patient with early relapse (*N* = 2 samples from two timepoints) following TKI discontinuation. Cells are colored based on manually annotated clusters (upper left UMAP), patients they were derived from (lower left UMAP), or scaled expressions of genes used to annotate the phenotypes (UMAPs on the right). **d** Differentially expressed genes (*P*_*adj*_ < 0.05, Bonferroni corrected *t*-test) between cluster 1 and cluster 0 from Fig. 6C. **e** Scaled average expressions (avg exp) and proportion of antigen-specific T cells in two patients expressing (pct.exp) canonical T cell markers. Anti-PR1 T cells were defined with tetramer staining and the anti-viral T cells were predicted with TCRGP against CMV, EBV, HSV2, and Influenza A. CML=chronic myeloid leukemia, CLL=chronic lymphocytic leukemia, AML= acute myeloid leukemia, RCC=renal cell carcinoma, NSCLC=non-small cell lung carcinoma, TKI=tyrosine kinase inhibitor. *n* refers to the number of patients and *N* to the number of samples where it differs from *n*. *=*P* < 0.05, **=*P* < 0.01, ***=*P* < 0.001, ****=*P* < 0.0001.

The phenotype of anti-PR1 was enriched to CD8 + T_EM/EMRA_ cells (odds-ratio OR 43.134), which were enriched in patients with CML before TKI treatment in comparison to healthy and other cancers (median 20.2% vs 5.37%, log_2_FC = 1.911, *P* = 0.0082, two-sided Mann-Whitney test, Fig. 6a, b). The anti-PR1 T cells themselves formed two clusters, where the cells from TFR patients were CD8 + T_EMRA/EFF_ phenotype with upregulated NK-associated genes (cluster 0, 93.10%) while anti-PR1 T cells from early relapse patient had more cells in a cluster of CD8 + T_EM_ phenotype (cluster 1, 43.60%) (Fig. 6c, d, DEGs in Supplementary Table 2). Importantly, these phenotypes were not seen in T cells specific to anti-viral antigens predicted by TCRGP (Fig. 6e).

As with the NK cells, the phenotype of anti-PR1 T cells treatment changed more in the patients with relapse than with TFR following TKI cessation. After TKI cessation in TFR patients, the anti-PR1 cells gained more cytotoxic phenotype which was not observed in the relapsing patient (6 m vs baseline *P* < 0.01, 12 m vs 6 m *P* < 0.0001, Mann-Whitney, Fig. 6e). In the early relapse patient, the genes associated with exhaustion (e.g., *HAVCR2/TIM3*, *PDCD1/PD1*) decreased, and genes associated with early exhaustion (e.g., *LAG3*, *TIGIT*) increased, reflecting the change in the clonal structure described above (Fig. 6e, Supplementary Fig. 12c). This was also accompanied by the upregulation of proliferation marker *MKI67*, costimulatory genes (*CD28*, *TNFRSF9* (4-1BB), and tumor-reactivity markers (e.g., *ENTPD1/CD39*, *ITGAE/CD103*) suggestive of an anti-tumor response in face of rising tumor levels (Fig. 6e). These changes were not seen in the anti-viral T cells (Fig. 6e).

## Discussion

In this study, we systematically compared the immune system in CML to healthy, other hematologic (AML, CLL) and solid (RCC, NSCLC) cancers and followed the immune responses in patients with CML discontinuing imatinib TKI treatment with different clinical outcomes. The immune cell populations that were the most distinctive in CML; NK cells and CD8 + T cells; were studied more thoroughly with in vitro co-culture and antigen-specific T cell sorting strategies.

Our analysis highlights the importance of NK cells in CML in an unsupervised manner: they were the most abundant immune cell cluster in CML and more expanded than in healthy or patients with other malignancies. In a more detailed NK cell phenotyping based on clustering, we found 7 distinct NK cell clusters that were consistent with previously published scRNAseq data from healthy donors [30–32]. Although the 7 different NK cell clusters were identified in healthy donors, a striking majority (median 92.2%) of their repertoire consisted of a CD56^dim^ cluster (cluster 2). The patients with CML had far more diverse NK cell repertoire than healthy, although the majority (60.7% in diagnosis) were annotated as active CD56^dim^ NK cells (cluster 0), with significantly upregulated expression of cytotoxic markers (e.g., *FCGR3A/CD16*, *GMZA/B/M/H*), cytokines (e.g., *CCL3/4*, *IFNG*), and activation markers (e.g., *TIGIT*). This active NK cell cluster was less abundant in the other cancers, including myeloid cancer AML, and its upregulated genes were also seen in the co-culture of NK cells with CML cells. In a comprehensive analysis of NK cells against 26 hematological cancer cell lines, myeloid cancers and especially CML cell line K562 resulted in a more activated phenotype [33], reflecting the active NK cell state found also in patients. As we observed prominent responses of NK cells also to the LAMA84 CML cell line, our findings suggest that NK cells may react particularly strongly towards CML cells. In fact, in a recent report, NK cells with hyperfunctional adaptive-like features were also found to be expanded in patients destined to good responses to imatinib in comparison to patients who did not [49].

The NK cell repertoire was altered by imatinib therapy and TKI cessation attempt. The active NK cells in patients with TFR upregulated genes found in the co-culture to be costimulatory, including *TNFRSF9*/*4-1BB*, *TNFRSF18*/*GITR*, *TNFRSF4/OX-40*, and *CRTAM*, in comparison to on TKI and relapse samples. Additionally, the TFR patients downregulated immune checkpoints *HAVCR2*/*TIM3* and *TIGIT* in comparison to relapse samples, indicating a more active state of NK cells in TFR. These data add to the importance of NK cells in controlling residual CML cells, which has been noted in several “stop TKI” trials, where their elevated number, maturation, and function, have been associated with a better chance of TFR [14–16].

In the tumor-immune cell analysis, the immune system had more predicted interactions with CD34+ cells expressing higher than lower BCR-ABL1 pathway activity. Thus, LSCs with low BCR-ABL1 activity may evade immune cell recognition, fitting with clinical findings showing minute BCR-ABL1 transcript levels for years in patients maintaining TFR [50]. Although the immune system may not eradicate the most primitive LSCs, it is able to control the disease, but this is not seen in all patients attempting TFR. The quiescent LSCs with low BCR-ABL1 activity had high upregulation of HLA class I/II and IFN-γ signature, which was found to be induced following NK cell engagement in the co-culture assay, indicating it as a possible immune evasion mechanism for NK cell killing. The most common inhibitory interactions between NK and CD34 + CML cells included *LGALS9* – *TIM3* and *PVR* and *TIGIT*, and the inhibitory ligands *LGALS9* and *PVR* were expressed in both *BCR-ABL1* low and high-expressing CD34+ cells. The upregulation of *LGALS9* and to some extent *PVR* in CML cells and their receptors *TIM3* and *TIGIT* in NK cells were observed in the co-culture of CML cell lines with NK cells. Interestingly, blocking of TIM3 in the co-culture assays also led to increased IFN-γ secretion although no clear effect on target cell killing was seen. A recent study identified elevated *HAVCR2*/TIM3 in CD8 + T cells from patients with relapse following TKI withdrawal [51] as we noted in our PR1-specific CD8 + T cells. As such, immune-checkpoint inhibitors targeting TIGIT and TIM3 are interesting molecular targets to induce more potent NK cell engagement against CML cells, but further studies are needed with larger cohort of primary CML cells. Several clinical trials targeting TIM3 are currently ongoing in different myeloid malignancies including myelodysplastic syndrome (MDS) and AML, but not currently in CML [52]. Also other immune-checkpoint inhibitors, like anti-LAG3, enhancing NK cells in addition to T cells are entering the clinic [53].

In addition to NK cells, the CD8 + T cells showed a distinct phenotype in CML in contrast to healthy and other cancers. We generated PR1-specific TCR-sequencing data from multiple CML patients but were not able to find exactly matching TCRs between the patients. However, we could detect amino acid level similarities between PR1-specific TCRs and thus were able to generate an in silico classifier with TCRGP [43] to detect anti-PR1 TCRs from previously unseen data with high accuracy. Previously, studies addressing antigen-specific responses have been limited by the time and sample-consuming techniques, thus warranting a need for novel computational methods which can then be applied for previously profiled TCRβ-seq and scRNA+TCRαβ-seq data [42, 43, 45–47, 54, 55].

Anti-PR1 CD8 + T cells occupied a larger part of the TCRβ-repertoire than any other studied antigen-specific T cell repertoire, like those against viruses (e.g., CMV, EBV, or Influenza A) or melanoma-associated antigens (MART1). Additionally, anti-PR1 T cells were also more abundant in CML than in healthy, although we noted that healthy individuals also harbor anti-PR1 T-cells as has been previously described [7]. However, the proportion of anti-MART1 T cells in melanoma was higher than anti-PR1 T cells in CML suggesting that not all tumor-associated antigens generate similar responses, warranting the search for other leukemia-associated antigens and their TCRs in CML. The anti-PR1 T cells were also more numerous and occupied a larger space of the repertoire in bone marrow than peripheral blood, which is in line with those in other cancers, where clonality is higher in the tumor microenvironment than in peripheral blood [44]. It should be also noted our prediction of anti-PR1 T cell responses from the larger cohort were not restricted to HLA-A*02:01, possibly generating false-positive findings.

However, scRNA+TCRαβ-seq data from HLA-A*02:01 + CML patients showed how the anti-PR1 T cell profile is highly cytotoxic in a patient who maintained the TFR, but larger study cohorts are needed to confirm these results. Also, patient with a quick relapse had a terminally exhausted phenotype of their anti-PR1 T cells, similarly to patients who did not benefit from donor lymphocyte infusion (DLI) harbored more terminally exhausted T cells [56]. Interestingly, dasatinib seemed to induce higher levels of anti-PR1 T cells than imatinib or other 2^nd^ generation TKIs, and these remained elevated after dasatinib withdrawal. Although dasatinib is known to induce large granular lymphocyte (LGL) clones and higher clonalities [19, 26, 57] via inhibition of activation-induced cell death [6], this was irrespective of clonality levels.

The limitations in our work include the use of peripheral blood samples for the immunological part of scRNA+TCRαβ-seq, as we focused on longitudinal samples and bone marrow sampling is not routine during clinical follow-up of CML. From the leukemic cells from the bone marrow, we were not able to assess the BCR-ABL1 status of the cells and relied on BCR-ABL1 score, which might miss some BCR-ABL1+ cells that are in the BCR-ABL1 low clusters. Also, the interactions were calculated between samples from peripheral blood and bone marrow, but we are confident that the main interactions (*PVR* – *TIGIT*, *LGALS9* – *HAVCR2*/TIM3) are not artificial as these interactions were supported by our in vitro experiments.

In conclusion, with the in-depth analysis of cellular and molecular immune responses in CML, we identified the active NK cells and anti-PR1 T cells that could help maintain TFR in patients discontinuing TKI treatment. Prospective collection of bone marrow samples in TKI discontinuation trials is warranted to elucidate the complex interactome between residual CML LSCs and immune subsets defined here helping more patients gain TFR.

## Methods

### CML patient samples

For the scRNA+TCRαβ-seq cohort, in total 13 patients were recruited, including 7 newly diagnosed, untreated chronic-phase CML patients from diagnosis and 6 CML patients that were treated with imatinib and underwent TKI discontinuation (Supplementary Table 1).

From the TKI stop patients, peripheral blood samples were collected at the time of discontinuation, 6 or 12 months after cessation, or at the time of relapse (patients *n* = 6, samples *N* = 20). Mononuclear cells were separated with Ficoll-Paque centrifugation and stored in liquid nitrogen.

From the diagnosis patients, peripheral blood samples were also collected from 4 newly-diagnosed untreated CML patients and mononuclear cells were separated and stored similarly. In addition, from 3 patients bone marrow samples were collected at diagnosis, and CD34+ cells were sorted with magnetic beads and stored in liquid nitrogen.

For the TCRβ-sequencing cohorts, in total 58 samples were recruited from patients enrolled for different CML treatment trials, and samples were collected from multiple time points and peripheral blood or bone marrow (Supplementary Table 1).

The study was approved by Helsinki University Central Hospital (HUCH) ethical committee. Written informed consent was received from all patients, and the study was conducted in accordance with the Declaration of Helsinki.

Data for the validation cohorts were acquired as stated in Supplementary Table 1.

### Single-cell RNA + TCRαβ sequencing of CML patients

Before single-cell sequencing, blood mononuclear cells were sorted with flow cytometry to enrich alive cells (sorting strategy shown in Supplementary Fig. 2a, b). From bone marrow CD34+ cells, both CD34+ sorted (*n* = 3 samples) and CD34+CD38neg sorted cells (*n* = 2 samples) were used.

Single cells were partitioned using a Chromium Controller (10X Genomics) and scRNA-seq and TCRαβ-libraries were prepared using Chromium Single Cell 5’ Library & Gel Bead Kit (10X Genomics), as per manufacturer’s instructions (CG000086 Rev D). Briefly, cells were suspended in 0.04% BSA in PBS and were loaded on the Chromium Single Cell A Chip. During the run, single-cell barcoded cDNA is generated in nanodroplet partitions. The droplets are subsequently reversed and the remaining steps are performed in bulk. Full-length cDNA was amplified using 14 cycles of PCR (Veriti, Applied Biosystems). TCR cDNA was further amplified in a hemi-nested PCR reaction using Chromium Single Cell Human T Cell V(D)J Enrichment Kit (10X Genomics). Finally, the total cDNA and the TCR-enriched cDNA were subjected to fragmentation, end repair and A-tailing, adaptor ligation, and sample index PCR (14 and 9 cycles, respectively).

The gene expression libraries were sequenced using an Illumina NovaSeq, S1 flowcell with the following read length configuration: Read1 = 26, i7 = 8, i5 = 0, Read2 = 91. The TCR-enriched libraries were sequenced using an Illumina HiSeq2500 in Rapid Run mode with the following read length configuration: Read1 = 150, i7 = 8, i5 = 0, Read2 = 150.

The raw data was processed using Cell Ranger 3.0.0 with GRCh38 as the reference genome with default parameters.

### NK cell expansion

NK cells were expanded for 14 days using K562-mbIL21-41BBL feeder cells as described previously [58]. On day 0, 5 million mononuclear cells, separated from a buffy coat with Ficoll-Paque gradient centrifugation, were suspended in 40 ml RPMI-1640 with 10% heat-inactivated fetal bovine serum (FBS), 2 mM L- glutamine, and 100 U/mL penicillin with 100 mg/mL streptomycin (PS) (referred to as R10), supplemented with 10 ng/ml recombinant human IL-2 (R&D Systems, 202-IL-050) together with 10 million irradiated (100 Gy) K562-mbIL21-41BBL feeder cells. After 7 days of culture and two passages, additional feeder cells were added in a 1:1 ratio. After 14 days of culture, NK cells were purified using an NK Cell Isolation Kit (Miltenyi) and frozen. Before the scRNAseq co-culture experiments, NK cells were thawed and cultured in R10 supplemented with 10 ng/ml recombinant human IL-2 (R&D Systems, 202-IL-050) 3 days before the experiments.

### Co-culture of expanded or primary NK cells with K562 or LAMA84 cell lines with multiplexed scRNAseq readout

K562 or LAMA84 cancer cells were obtained from DSMZ (German Collection of Microorganisms and Cell Cultures) and co-cultured at 500,000 cells/well on a 24-well plate with either expanded NK cells (1:4 effector-to-target ratio), NK cells directly extracted from mononuclear cells from the same donor (1:2 effector-to-target ratio) or only R10 culture medium (targets only) were added, resulting in a total volume of 1 ml R10. The cell lines were tested for mycoplasma contamination biweekly.

After 24 h at 37°C and 5% CO2, cells from each well were washed 3 times with 10 ml PBS and resuspended in 100 µl Cell Staining Buffer (BioLegend). Additionally, 10 µl TruStain FcX blocking reagent (BioLegend) was added, and cells were incubated for 10 min. A unique TotalSeq-A hashing antibody (BioLegend) was added to each sample (1–2 µl/1–2 µg per sample) and cells were incubated at +4 °C for 30 min covered from light. Cells were then washed 5 times with 3 ml staining buffer and samples were combined in 1 ml staining buffer, centrifuged, resuspended in PBS + 0.04% bovine serum albumin (BSA), and proceeded to scRNAseq.

The Chromium Single Cell 3’ RNAseq run and library preparations were done using the 10x Genomics Chromium Next GEM Single Cell 3’ Gene Expression version 3.1 Dual Index chemistry with the modifications described in Stoeckius et al. [59], https://cite-seq.com/, and according to the slightly improved protocol described in www.biolegend.com/enus/protocols/totalseq-a-antibodies-and-cell-hashing-with10x-single-cell-3-reagent-kit-v3-3-1-protocol. The 3’ GEX and Cell Hashing (multiplexing) libraries were sequenced using Illumina NovaSeq 6000 system using read lengths: 28 bp (Read 1), 10 bp (i7 Index), 10 bp (i5 Index) and 90 bp (Read 2).

### TCRβ-sequencing

TCRβ-sequencing from genomics DNA was conducted as previously described with ImmunoSEQ assay by Adaptive Biotechnologies Corp [60].

### HLA typing

All the samples were typed at the Histocompatibility Testing Laboratory, Finnish Red Cross Blood Service accredited by European Federation for Immunogenetics. The HLA specificities were reported based on the current World Health Organization (WHO) nomenclature for the HLA system. The typing for HLA-A, -B, -C, and -DRB1 loci was performed using the Luminex bead array technology together with sequence-specific oligonucleotide probes (Commercial LabType kits RSSO1A, RSSO1B, RSSO1C, RSSO2B1, One Lambda, Los Angeles, CA). The bead array data were interpreted according to the manufacturer’s recommendations using the HLA Fusion software 3.2 (One Lambda). A proportion of samples was further typed by the Sanger sequencing method to obtain higher resolution for the HLA type (Commercial AlleleSEQR kits 08K60-06, 08K61-06, 08K62-06, 08K63-06, GenDx, Utrecht, Netherlands). The sequencing data were analyzed using the SBTengine software 3.9.0.2563 (GenDx).

### pMHC PR1-multimer sorting

Peripheral blood mononuclear cells (PBMCs) were obtained from 11 blood samples from CML patients with known HLA type at HLA-A, HLA-B, and HLA-DR loci and known CMV antibody status from a serological test. Cells were stained with fluorophore-conjugated pMHC tetramer: Biotin-labeled Pro5 MHC Pentamer HLA-A*02:01 epitope VLQELNVTV Code: F250-1A-E Batch: MP/5410-06, ProImmune.

### Co-culture of expanded NK cells with K562 or LAMA84 cell lines for flow cytometry-based cytotoxicity assay

Anti-TIM3 blocking antibody, sabatolimab (MCE, HY-P99044), or IgG4 kappa (MCE, HY-P99003) was added to expanded NK cells (20,000/well) on 96 well plate for 20 min on +37 °C. K562 or LAMA84 cancer cells were added at 40,000 cells/well (0.5:1 effector-to-target ratio). R10 with GolgiStop (BD, 554724) and anti-CD107a antibody (BD, 5558000) were added. And final volume of 100 µl/well was obtained with R10 addition. Plates were incubated overnight at +37 °C and 5% CO2.

Plates were washed with PBS-EDTA-BSA and resuspended to PBS-EDTA-BSA containing fluorochrome conjugated surface antibodies; CD3 (BD, 332771, 5 µl/well), CD56 (BD, 345812, 2 µl/well), NKG2D (BD, 562365, 0.25 µl/well). After 15 min incubation in dark, plates were washed, and incubated 20 min at +4 °C with BD Cytofix/Cytoperm^TM^ Fixation and permeabilization solution (554714). Plates were washed with BD Perm/Wash^TM^ buffer (554714) and incubated 30 min at dark with intracellular antibodies IFNg (BD, 554702, 0.5 µl/well) and TNFa (BD, 561311, 0.5 µl/well) in PBSA-EDTA-BSA. After washing plates were measured with Novocyte Quanteon.

### Viability assay for co-culture

ONE-Glo™ Luciferase assay (Promega, E6130) was performed to monitor cell viability after co-culture. Co-culture plates without GolgiStop and anti-CD107a were incubated over night at +37 °C and 5% CO2. Assay was performed following manufacturer’s instructions and plated were measured with FluoStar Omega microplate reader.

### Single-cell RNA-sequencing data analysis

All cells, profiled in the study gathered from publications (Supplementary Table 1) were subject to the same quality control. Cells with high amount of mitochondrial transcripts (>15% of all UMI counts) or ribosomal transcripts (>50%), cells with low or high (<100 or >4,500 genes) the number of genes expressed, cells expressing low or high (<25% or >60%) amount of house-keeping genes or cells with low or high read depth (<500 or >30 000) were excluded from the analyses.

To overcome the batch effect from different studies and samples, we used a probabilistic framework to overcome different nuisance factors of variation in an unsupervised manner with the deep generative modeling tool scVI (0.5.0) [27]. Briefly, the transcriptome of each cell is encoded through a nonlinear transformation into a low-dimensional, batch-corrected latent embedding. Data were visualized with the UMAP-dimensionality reduction [61], calculated with the RunUMAP-function implemented in Seurat (4.0.4) [62] on the latent embeddings.

In a cluster-agnostic analysis, we predicted the cell types with Celltypist (v.1.2.0), which is essentially a logistic regression classifier that works with a built-in database of previously annotated RNA-sequencing data. We used the default parameters with the previously trained “Immune_All_Low.pkl” model available with the model. We validated the cell type predictions with manual inspection of differentially expressed genes between the cell types, expression of canonical markers, Euclidean distances to other clusters, signature scores, TCR-repertoire, and with automated cell type annotation with SingleR [29] (1.2.4) based on sorted immune subsets with default parameters.

Additionally, we performed a cluster-based analysis, where the latent embeddings were then used for graph-based clustering implemented in Seurat (4.0.4) [62]. The optimal number of clusters was determined with knee-plots on the used clustering resolution parameter as a function of the number of clusters, where the optimal number of clusters was determined as where the number of clusters first plateaued. Clusters were then annotated by analysis of manual inspection of differentially expressed genes between the cell types, expression of canonical markers, Euclidean distances to other clusters, signature scores, and TCR-repertoire analyses.

Differential expression analyses were performed based on the t-test, as suggested by Soneson et al. [63]. Enrichment analysis was done by using hypergeometric testing on GO and HALLMARK-categories from R-package ClusterProfiler [64].

Different scores were calculated with Seurat’s AddModuleScore-function, which is an implementation of the method suggested by Tirosh et al. [65]. The genes used to calculate the cytotoxicity score included *IFNG*, GZMA, *GZMB, GZMM, GZMH, PRF1*, and *GNLY* [66]; the exhaustion score included *CTLA4*, *LAG3*, *PDCD1*, *HAVCR2*, and *TIGIT*; HLA class I score included *HLA-A, HLA-B*, and *HLA-C*; HLA class II score included *HLA-DMA, HLA-DMB, HLA-DOA, HLA-DOB, HLA-DPB1, HLA-DPA1, HLA-DQA1, HLA-DQA2, HLA-DQB1, HLA-DQB2, HLA-DRA, HLA-DRB1*, and *HLA-DRB5*. The genes used to calculate the BCR-ABL1 score included differentially expressed genes between BCR-ABL1+ and BCR-ABL1- cells found by Gisutacchini et al. [37], including genes previously implicated with CML (*CD33, CD69, SELL, CXCR4, CDC42, MMRN1, ANZA2, CDK6, GAS2, IFITM1, CTNNB1, NFKBIA, FOS, NCF4, MGST2, TIMP1, EZH2*) [37] and novel candidate genes (*LGALS1, RXCFP1, AREG, RAB31, PRSS21, TUBB6, YBX1, CKS2, PTPBP3, PSIP1, CDCA7, MT2A, FHL1, LAMTOR2, S100A10, LAPTM4B, DCTPP1, STON2, SRSF2, HAT1, LOP2, PCDH9*) with various implications related to cell adhesion, cell signaling, protein binding, inflammatory response, and mRNA splicing.

Ligand-receptor interactions were calculated with CellPhoneDB (2.0.0) with default parameters [38] on subsampled cells from each cell type to have the identical amount of cells for each subtype.

Demultiplexing of the co-culture assay was performed with the kernel-density estimation of centered log-ratio-normalized hashtag-oligo (HTO) UMI counts, which essentially estimates whether a singular HTO is expressed more than others. Bandwidth for the kernel-density estimation is estimated through biased cross-validation, after which the hashtag with the highest expression is omitted from the probability estimation. The predictions were cross-referenced to the HTODemux function in Seurat. Only cells where expression of a singular HTO was identified (“singlets”) were considered for further analyses.

### TCR sequencing and data analysis

TCR sequencing was performed with the ImmunoSEQ platform (Adaptive Biotechnologies). Analyses started with the TCRβ matrices provided by the Adaptive Biotechnologies preprocessing pipeline. All data were analyzed with VDJtools [67] (ver 1.2.1) or R (ver 4.0.2) and thus were transformed to VDJtools-format to reduce the complexity of the data. Non-productive clonotypes were removed from the analysis. As the healthy control data was sequenced deeper than our CML cohort, we used a minimum sampling depth of 40,000 reads per sample for the health data and subsampled all samples with more reads to 40,000 reads to normalize samples to remove biases for depth-dependent statistics. Multiple different diversity metrics, including Shannon-Wiener, Simpson, and clonality indexes were calculated with CalcDiversityStats-function on both unsampled and subsampled repertoire data.

For unsupervised detection of clonotypes that shared amino acid level features, the web server for GLIPH2 [42] was used with CD8 as the reference set. For supervised detection of epitope-specific clonotypes, TCRGP for PR1-specific data was trained using 10-fold cross-validation with default parameters with 2000 iterations and a learning rate of 0.005, and the other models were downloaded from the original publication [43].

### Statistical testing

*P*-values were calculated with nonparametric tests, including Mann–Whitney test (two groups), Kruskal–Wallis test (more than two groups), and Fisher’s exact test where the alternative hypotheses are reported. *P*-values were corrected with Bonferroni (differentially expressed genes) or with Benjamini–Hochberg (all other tests) adjustment. All calculations were done with R (4.0.2) or Python (3.7.4). In the box plots, the center line corresponds to the median, the box corresponds to the interquartile range (IQR), and whiskers 1.5 × IQR, while outlier points are plotted individually where present.

### Supplementary information

Supplementary Figures and Supplementary Data legends

Supplementary Data 1

Supplementary Data 2

Supplementary Data 3

Supplementary Data 4

## Data Availability

The raw scRNA+TCRαβ-seq data generated in this study are available in the European Genome-Phenome Archive under accession code EGAS00001005044. The processed scRNA+TCRαβ-seq data and the Seurat-objects are available at Zenodo under 10.5281/zenodo.7330586. The bone marrow CML cell scRNA-seq data is available GSE236233 [68]. The processed TCRβ-seq data, including the PR1-classifier for TCRGP, are available at Zenodo under 10.5281/zenodo.7330586. All the data are within restricted access due to GDPR and data can be accessed by placing a request which will be reviewed promptly. The publicly available scRNA+TCRαβ-sequencing and TCRβ-sequencing data used in this study are listed in Supplementary Table 1.

## Code availability

The code to reproduce the key findings is available at https://github.com/janihuuh/cml_stop_manu.

## References

[1] Mahon FX, Réa D, Guilhot J, Guilhot F, Huguet F, Nicolini F (2010). Discontinuation of imatinib in patients with chronic myeloid leukaemia who have maintained complete molecular remission for at least 2 years: the prospective, multicentre Stop Imatinib (STIM) trial. Lancet Oncol.

[2] Ross DM, Branford S, Seymour JF, Schwarer AP, Arthur C, Yeung DT (2013). Safety and efficacy of imatinib cessation for CML patients with stable undetectable minimal residual disease: results from the TWISTER study. Blood.

[3] Saussele S, Richter J, Guilhot J, Gruber FX, Hjorth-Hansen H, Almeida A (2018). Discontinuation of tyrosine kinase inhibitor therapy in chronic myeloid leukaemia (EURO-SKI): a prespecified interim analysis of a prospective, multicentre, non-randomised, trial. Lancet Oncol.

[4] Mori S, Vagge E, le Coutre P, Abruzzese E, Martino B, Pungolino E (2015). Age and dPCR can predict relapse in CML patients who discontinued imatinib: the ISAV study. Am J Hematol.

[5] Nicolini FE, Dulucq S, Guilhot J, Etienne G, Mahon F-X (2018). The Evaluation of Residual Disease By Digital PCR, and TKI Duration Are Critical Predictive Factors for Molecular Recurrence after for Stopping Imatinib First-Line in Chronic Phase CML Patients: Results of the STIM2 Study. Blood.

[6] Huuhtanen J, Ilander M, Yadav B, Dufva OM, Lähteenmäki H, Kasanen T et al. IFN-α with dasatinib broadens the immune repertoire in patients with chronic-phase chronic myeloid leukemia. *J Clin Invest* 2022; **132**. 10.1172/JCI152585. 10.1172/JCI152585 PMC9433106 36047494

[7] Molldrem JJ, Lee PP, Wang C, Felio K, Kantarjian HM, Champlin RE (2000). Evidence that specific T lymphocytes may participate in the elimination of chronic myelogenous leukemia. Nat Med.

[8] Hughes A, Yong ASM (2017). Immune effector recovery in chronic myeloid leukemia and treatment-free remission. Front Immunol.

[9] Brück O, Blom S, Dufva O, Turkki R, Chheda H, Ribeiro A (2018). Immune cell contexture in the bone marrow tumor microenvironment impacts therapy response in CML. Leukemia.

[10] Kreutzman A, Yadav B, Brummendorf TH, Gjertsen BT, Hee Lee M, Janssen J (2019). Immunological monitoring of newly diagnosed CML patients treated with bosutinib or imatinib first-line. OncoImmunology.

[11] Hsieh Y-C, Kirschner K, Copland M (2021). Improving outcomes in chronic myeloid leukemia through harnessing the immunological landscape. Leukemia.

[12] Corbin AS, Agarwal A, Loriaux M, Cortes J, Deininger MW, Druker BJ (2011). Human chronic myeloid leukemia stem cells are insensitive to imatinib despite inhibition of BCR-ABL activity. J Clin Invest.

[13] Hamilton A, Helgason GV, Schemionek M, Zhang B, Myssina S, Allan EK (2012). Chronic myeloid leukemia stem cells are not dependent on Bcr-Abl kinase activity for their survival. Blood.

[14] Ilander M, Olsson-Strömberg U, Schlums H, Guilhot J, Brück O, Lähteenmäki H (2017). Increased proportion of mature NK cells is associated with successful imatinib discontinuation in chronic myeloid leukemia. Leukemia.

[15] Rea D, Henry G, Khaznadar Z, Etienne G, Guilhot F, Nicolini F (2017). Natural killer-cell counts are associated with molecular relapse-free survival after imatinib discontinuation in chronic myeloid leukemia: the IMMUNOSTIM study. Haematologica.

[16] Imagawa J, Tanaka H, Okada M, Nakamae H, Hino M, Murai K (2015). Discontinuation of dasatinib in patients with chronic myeloid leukaemia who have maintained deep molecular response for longer than 1 year (DADI trial): a multicentre phase 2 trial. Lancet Haematol.

[17] Kumagai T, Nakaseko C, Nishiwaki K, Yoshida C, Ohashi K, Takezako N et al. Silent NK/T cell reactions to dasatinib during sustained deep molecular response before cessation are associated with longer treatment‐free remission. *Cancer Science* 2020; 1–12. 10.1111/cas.14518 PMC7419041 32614159

[18] Irani YD, Hughes A, Clarson J, Kok CH, Shanmuganathan N, White DL (2020). Successful treatment-free remission in chronic myeloid leukaemia and its association with reduced immune suppressors and increased natural killer cells. Br J Haematol.

[19] Mustjoki S, Ekblom M, Arstila TP, Dybedal I, Epling-Burnette PK, Guilhot F (2009). Clonal expansion of T/NK-cells during tyrosine kinase inhibitor dasatinib therapy. Leukemia.

[20] Wu TD, Madireddi S, de Almeida PE, Banchereau R, Chen Y-JJ, Chitre AS (2020). Peripheral T cell expansion predicts tumour infiltration and clinical response. Nature.

[21] Rendeiro AF, Krausgruber T, Fortelny N, Zhao F, Penz T, Farlik M (2020). Chromatin mapping and single-cell immune profiling define the temporal dynamics of ibrutinib response in CLL. Nat Commun.

[22] Emerson RO, DeWitt WS, Vignali M, Gravley J, Hu JK, Osborne EJ (2017). Immunosequencing identifies signatures of cytomegalovirus exposure history and HLA-mediated effects on the T cell repertoire. Nat Genet.

[23] Wang VE, Blaser BW, Patel RK, Behbehani GK, Rao AA, Durbin-Johnson B (2021). Inhibition of MET Signaling with Ficlatuzumab in Combination with Chemotherapy in Refractory AML: Clinical Outcomes and High-Dimensional Analysis. Blood Cancer Discov.

[24] Penter L, Gohil SH, Lareau C, Ludwig LS, Parry EM, Huang T (2021). Longitudinal Single-Cell Dynamics of Chromatin Accessibility and Mitochondrial Mutations in Chronic Lymphocytic Leukemia Mirror Disease History. Cancer Discov.

[25] Datasets -Single Cell Gene Expression -Official 10× Genomics Support. https://support.10xgenomics.com/single-cell-gene-expression/datasets/2.1.0/pbmc8k? (accessed 27 Jul2020).

[26] Huuhtanen J, Bhattacharya D, Lönnberg T, Kankainen M, Kerr C, Theodoropoulos J (2022). Single-cell characterization of leukemic and non-leukemic immune repertoires in CD8+ T-cell large granular lymphocytic leukemia. Nat Commun.

[27] Lopez R, Regier J, Cole MB, Jordan MI, Yosef N (2018). Deep generative modeling for single-cell transcriptomics. Nat Methods.

[28] Domínguez Conde C, Xu C, Jarvis LB, Rainbow DB, Wells SB, Gomes T (2022). Cross-tissue immune cell analysis reveals tissue-specific features in humans. Science.

[29] Aran D, Looney AP, Liu L, Wu E, Fong V, Hsu A (2019). Reference-based analysis of lung single-cell sequencing reveals a transitional profibrotic macrophage. Nat Immunol.

[30] Yang C, Siebert JR, Burns R, Gerbec ZJ, Bonacci B, Rymaszewski A (2019). Heterogeneity of human bone marrow and blood natural killer cells defined by single-cell transcriptome. Nat Commun.

[31] Pfefferle A, Netskar H, Ask EH, Lorenz S, Goodridge JP, Sohlberg E et al. A Temporal Transcriptional Map of Human Natural Killer Cell Differentiation. *bioRxiv*. 2019; 630657.

[32] Smith SL, Kennedy PR, Stacey KB, Worboys JD, Yarwood A, Seo S (2020). Diversity of peripheral blood human NK cells identified by single-cell RNA sequencing. Blood Adv.

[33] Dufva O, Gandolfi S, Huuhtanen J, Dashevsky O, Saeed K, Klievink J et al. Single-cell functional genomics of natural killer cell evasion in blood cancers. bioRxiv. 2022; 2022.08.22.504722.

[34] Warfvinge R, Geironson L, Sommarin MNE, Lang S, Karlsson C, Roschupkina T (2017). Single-cell molecular analysis defines therapy response and immunophenotype of stem cell subpopulations in CML. Blood.

[35] van Galen P, Hovestadt V, Wadsworth Ii MH, Hughes TK, Griffin GK, Battaglia S (2019). Single-Cell RNA-Seq Reveals AML Hierarchies Relevant to Disease Progression and Immunity. Cell.

[36] Dhapola P, Eldeeb M, Ugale A, Olofzon R Nabo–a framework to define leukemia-initiating cells and differentiation in single-cell RNA-sequencing data. *bioRxiv* 2020.https://www.biorxiv.org/content/10.1101/2020.09.30.321216.abstract.

[37] Giustacchini A, Thongjuea S, Barkas N, Woll PS, Povinelli BJ, Booth CAG (2017). Single-cell transcriptomics uncovers distinct molecular signatures of stem cells in chronic myeloid leukemia. Nat Med.

[38] Efremova M, Vento-Tormo M, Teichmann SA, Vento-Tormo R (2020). CellPhoneDB: inferring cell–cell communication from combined expression of multi-subunit ligand–receptor complexes. Nat Protoc.

[39] Schwartz S, Patel N, Longmire T, Jayaraman P, Jiang X, Lu H (2022). Characterization of sabatolimab, a novel immunotherapy with immuno-myeloid activity directed against TIM-3 receptor. Immunother Adv.

[40] Gleason MK, Lenvik TR, McCullar V, Felices M, O’Brien MS, Cooley SA (2012). Tim-3 is an inducible human natural killer cell receptor that enhances interferon gamma production in response to galectin-9. Blood.

[41] Burchert A, Wölfl S, Schmidt M, Brendel C, Denecke B, Cai D (2003). Interferon-alpha, but not the ABL-kinase inhibitor imatinib (STI571), induces expression of myeloblastin and a specific T-cell response in chronic myeloid leukemia. Blood.

[42] Huang H, Wang C, Rubelt F, Scriba TJ, Davis MM (2020). Analyzing the Mycobacterium tuberculosis immune response by T-cell receptor clustering with GLIPH2 and genome-wide antigen screening. Nat Biotechnol.

[43] Jokinen E, Huuhtanen J, Mustjoki S, Heinonen M, Lähdesmäki H (2021). Predicting recognition between T cell receptors and epitopes with TCRGP. PLoS Comput Biol.

[44] Huuhtanen J, Chen L, Jokinen E, Kasanen H, Lönnberg T, Kreutzman A (2022). Evolution and modulation of antigen-specific T cell responses in melanoma patients. Nat Commun.

[45] Jokinen E, Dumitrescu A, Huuhtanen J, Gligorijević V, Mustjoki S, Bonneau R et al. TCRconv: Predicting recognition between T cell receptors and epitopes using contextualized motifs. *Bioinformatics* 2022. 10.1093/bioinformatics/btac788. 10.1093/bioinformatics/btac788 PMC9825763 36477794

[46] Dash P, Fiore-Gartland AJ, Hertz T, Wang GC, Sharma S, Souquette A (2017). Quantifiable predictive features define epitope-specific T cell receptor repertoires. Nature.

[47] Greiff V, Yaari G, Cowell LG (2020). Mining adaptive immune receptor repertoires for biological and clinical information using machine learning. Curr Opin Syst Biol.

[48] Glanville J, Huang H, Nau A, Hatton O, Wagar LE, Rubelt F (2017). Identifying specificity groups in the T cell receptor repertoire. Nature.

[49] Krishnan V, Schmidt F, Nawaz Z, Venkatesh PN, Lee KL, Ren X (2023). A single-cell atlas identifies pretreatment features of primary imatinib resistance in chronic myeloid leukemia. Blood.

[50] Ross DM, Pagani IS, Shanmuganathan N, Kok CH, Seymour JF, Mills AK (2018). Long-term treatment-free remission of chronic myeloid leukemia with falling levels of residual leukemic cells. Leukemia.

[51] Irani YD, Kok CH, Clarson J, Shanmuganathan N, Branford S, Yeung DT (2023). Association of TIM-3 checkpoint receptor expression on T cells with treatment-free remission in chronic myeloid leukemia. Blood Adv.

[52] Wolf Y, Anderson AC, Kuchroo VK (2020). TIM3 comes of age as an inhibitory receptor. Nat Rev Immunol.

[53] Huuhtanen J, Kasanen HH, Peltola K, Lönnberg T, Glumoff V, Brück O et al. Single-cell characterization of anti-LAG3+anti-PD1 treatment in melanoma patients. *J Clin Invest* 2023. 10.1172/JCI164809. 10.1172/JCI164809 PMC10014104 36719749

[54] Brown AJ, Snapkov I, Akbar R, Pavlović M, Miho E, Sandve GK (2019). Augmenting adaptive immunity: Progress and challenges in the quantitative engineering and analysis of adaptive immune receptor repertoires. Mol Syst Des Eng.

[55] Cowell LG (2020). The diagnostic, prognostic, and therapeutic potential of adaptive immune receptor repertoire profiling in cancer. Cancer Res.

[56] Bachireddy P, Azizi E, Burdziak C, Nguyen VN, Ennis CS, Maurer K (2021). Mapping the evolution of T cell states during response and resistance to adoptive cellular therapy. Cell Rep.

[57] Mustjoki S, Auvinen K, Kreutzman A, Rousselot P, Hernesniemi S, Melo T (2013). Rapid mobilization of cytotoxic lymphocytes induced by dasatinib therapy. Leukemia.

[58] Denman CJ, Senyukov VV, Somanchi SS, Phatarpekar PV, Kopp LM, Johnson JL (2012). Membrane-bound IL-21 promotes sustained ex vivo proliferation of human natural killer cells. PLoS One.

[59] Stoeckius M, Zheng S, Houck-Loomis B, Hao S, Yeung BZ, Mauck WM (2018). Cell Hashing with barcoded antibodies enables multiplexing and doublet detection for single cell genomics. Genome Biol.

[60] Carlson CS, Emerson RO, Sherwood AM, Desmarais C, Chung M-W, Parsons JM (2013). Using synthetic templates to design an unbiased multiplex PCR assay. Nat Commun.

[61] Becht E, McInnes L, Healy J, Dutertre C-A, Kwok IWH, Ng LG (2018). Dimensionality reduction for visualizing single-cell data using UMAP. Nat Biotechnol.

[62] Hao Y, Hao S, Andersen-Nissen E, Mauck WM, Zheng S, Butler A (2021). Integrated analysis of multimodal single-cell data. Cell.

[63] Soneson C, Robinson MD (2018). Bias, robustness and scalability in single-cell differential expression analysis. Nat Methods.

[64] Yu G, Wang L-G, Han Y, He Q-Y (2012). clusterProfiler: an R package for comparing biological themes among gene clusters. OMICS.

[65] Tirosh I, Izar B, Prakadan SM, Wadsworth MH, Treacy D, Trombetta JJ (2016). Dissecting the multicellular ecosystem of metastatic melanoma by single-cell RNA-seq. Science.

[66] Dufva O, Pölönen P, Brück O, Keränen MAI, Klievink J, Mehtonen J (2020). Immunogenomic Landscape of Hematological Malignancies. Cancer Cell.

[67] Shugay M, Bagaev DV, Turchaninova MA, Bolotin DA VDJtools : Unifying Post-analysis of T Cell Receptor Repertoires. 2015;1–16. 10.1371/journal.pcbi.1004503 PMC4659587 26606115

[68] Warfvinge R, Ulfsson LG, Dhapola P, Safi F, Sommarin MNE, Soneji S et al. Single cell multi-omics analysis of chronic myeloid leukemia links cellular heterogeneity to therapy response. *bioRxiv*. 2023; 2023.08.16.553504.

