# Supplementary material for: Single-cell analysis of immune recognition in chronic myeloid leukemia patients following tyrosine kinase inhibitor discontinuation
Source: Leukemia. 2023 Nov 2;38(1):109–25. doi: 10.1038/s41375-023-02074-w (PMC10776410; doi:10.1038/s41375-023-02074-w)
Supplement: Supplementary file 1 — Supplementary Figures and Supplementary Data legends [file 41375_2023_2074_MOESM1_ESM.pdf]

Supplementary Table 1: Clinical characteristics
Supplementary Table 2: Differentially expressed genes, pathways, and abundances
Supplementary Table 3: Significant ligand-receptor pairs
Supplementary Table 4: Antigen specificity results related to PR1-specific TCRs

Supplementary Figure 1

a

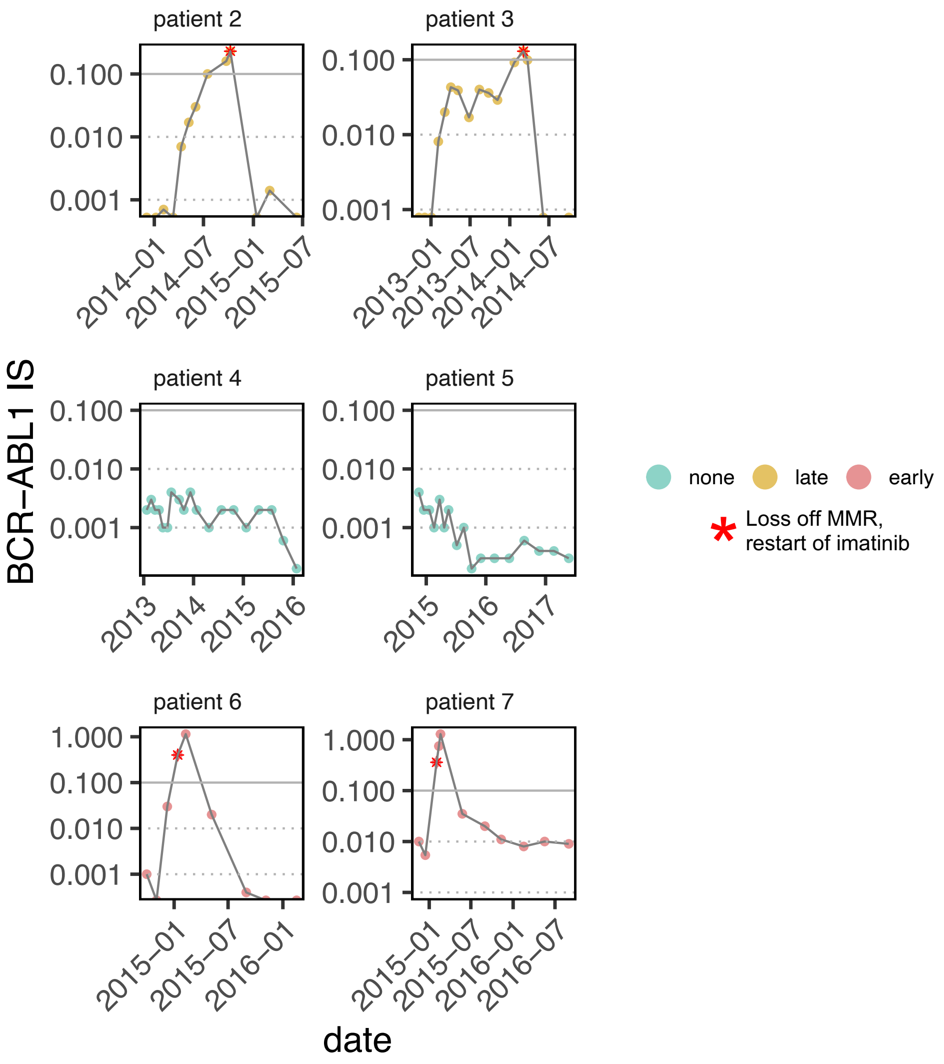

Supplementary Figure 1: BCR-ABL1-transcript levels

a) The amount of BCR-ABL1-transcript in the international scale (IS) following TKI cessation in the samples profiled in the study. Per study protocol, patients restarted imatinib after the loss of major molecular remission (MMR) and deep molecular remission was regained. From all patients samples were collected at the time of imatinib discontinuation. From patients staining in remission, follow-up samples were collected at 6 and 12 months. From late relapsing patients samples were collected at 6 months and at the time of relapse (around 12 months). From early relapse patients, the only follow-up sample was collected at the time of relapse which occurred around 3 months.

Supplementary Figure 2

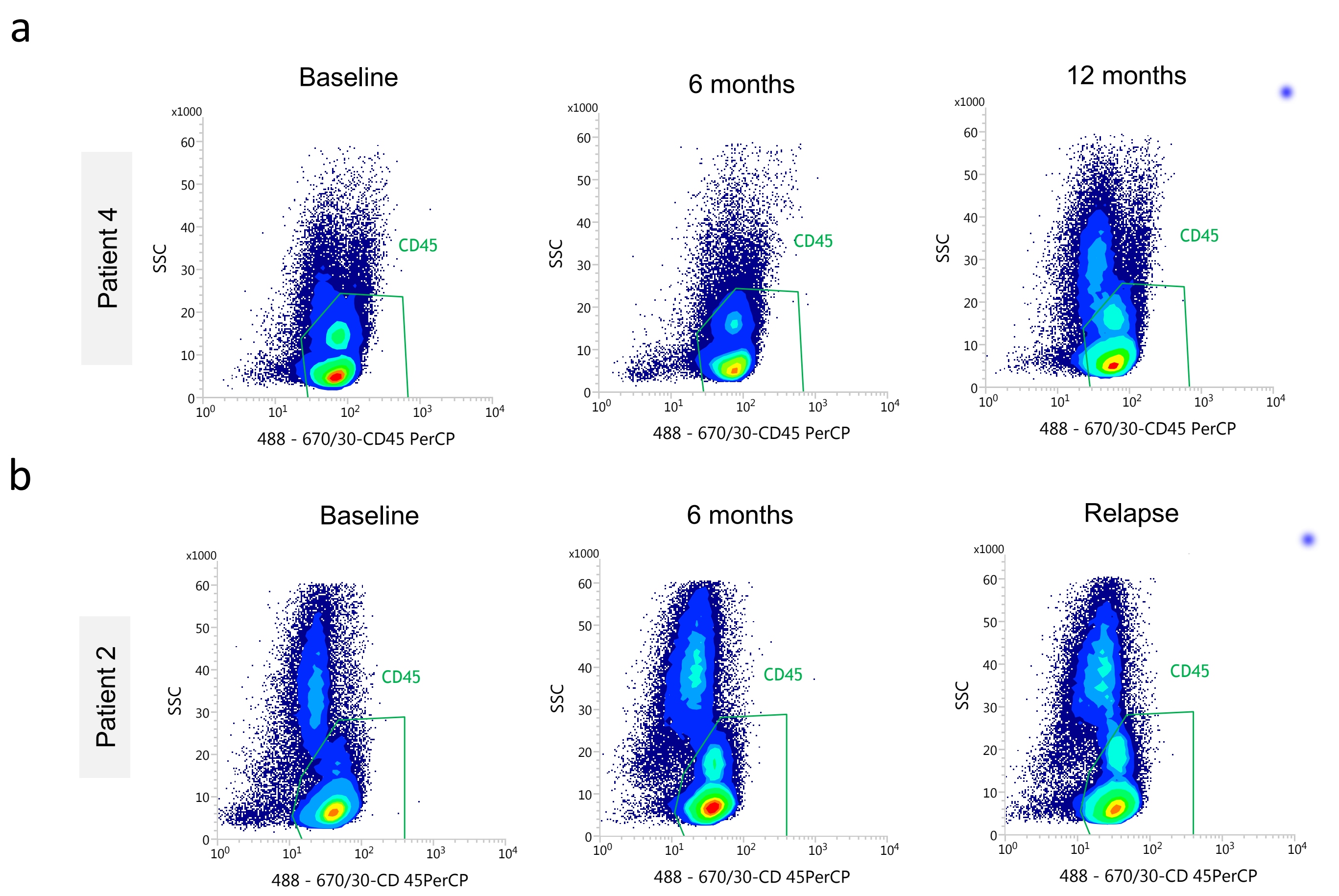

Supplementary Figure 2: FACS sorting strategies for single-cell RNA-sequencing

a) Scatter plot showing the CD45+ sorting strategy used to isolate the blood mononuclear cells for the single-cell profiling. Sorted cells include the CD45+ lymphocyte and monocyte population. Sample from patient 2 at baseline before TKI cessation and after 6 and 12 months following cessation.

b) Similar plot as in panel **a)** but from patient 4 at baseline, 6 months following TKI cessation and at relapse.

Supplementary Figure 3

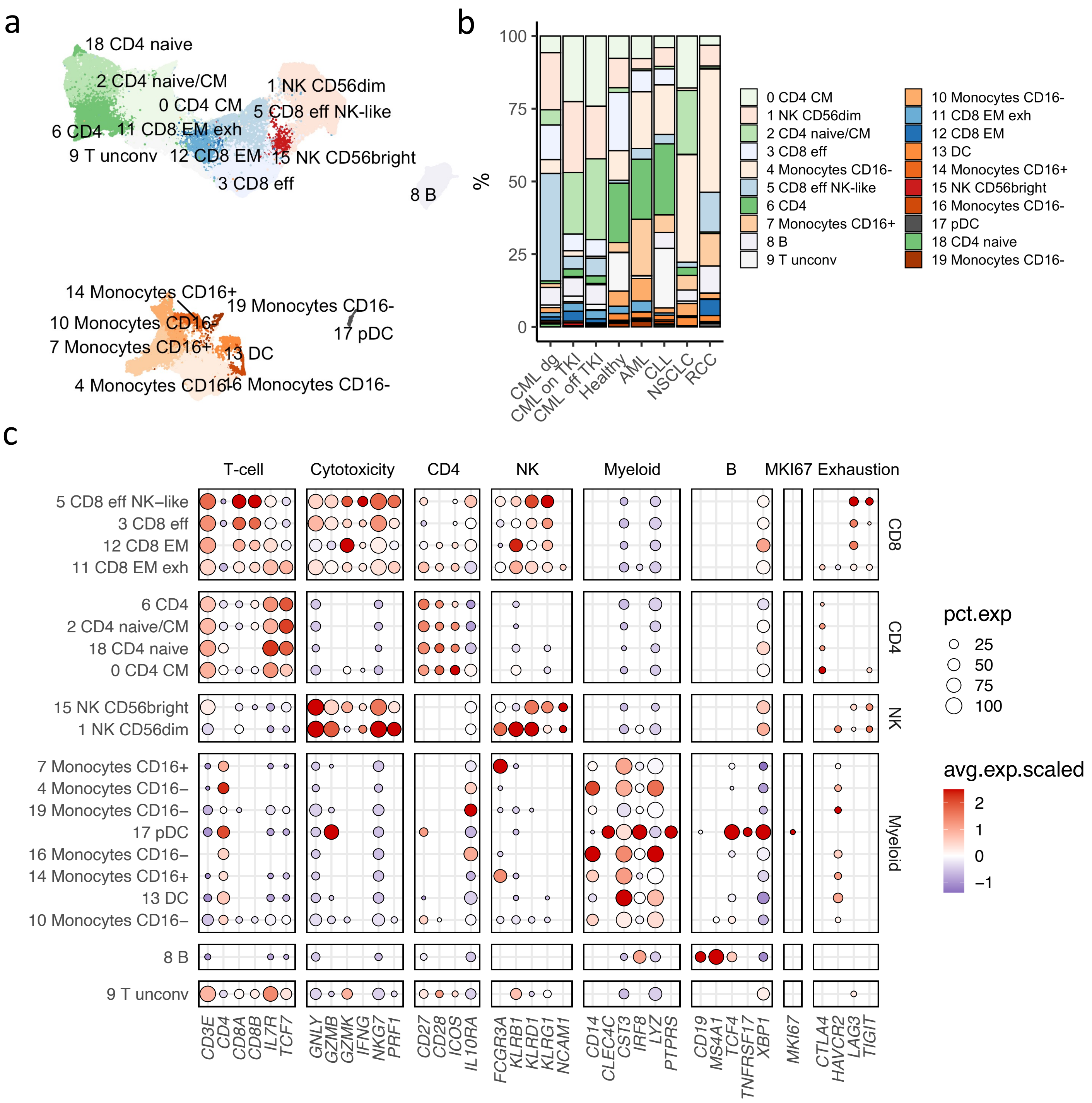

Supplementary Figure 3: Clustering and manual annotation of scRNAseq data

a) Uniform Manifold Approximation and Projection (UMAP) representation of the pooled RNA profiles of 247,946 CD45+ single-cells including peripheral blood samples from patients with CML (diagnosis *n*=4, on TKI *n*=6, *N*=6, off TKI *n*=6, *N*=10), patients with untreated hematological cancers (CLL *n*=13, AML *n*=11), patients with untreated solid cancer (RCC *n*=3, NSCLC *n*=1), and healthy controls (*n*=7). Cells are colored based on manually annotated clusters.

b) Median proportion of cells within each condition.

c) Scaled average expressions (avg exp) and proportion of cells expressing (pct.exp) the canonical markers used to define the clusters. Encircled dots are differentially expressed (P_{adj}<0.05, Bonferroni corrected *t*-test) in given cluster in comparison to other clusters.

Supplementary Figure 4

a

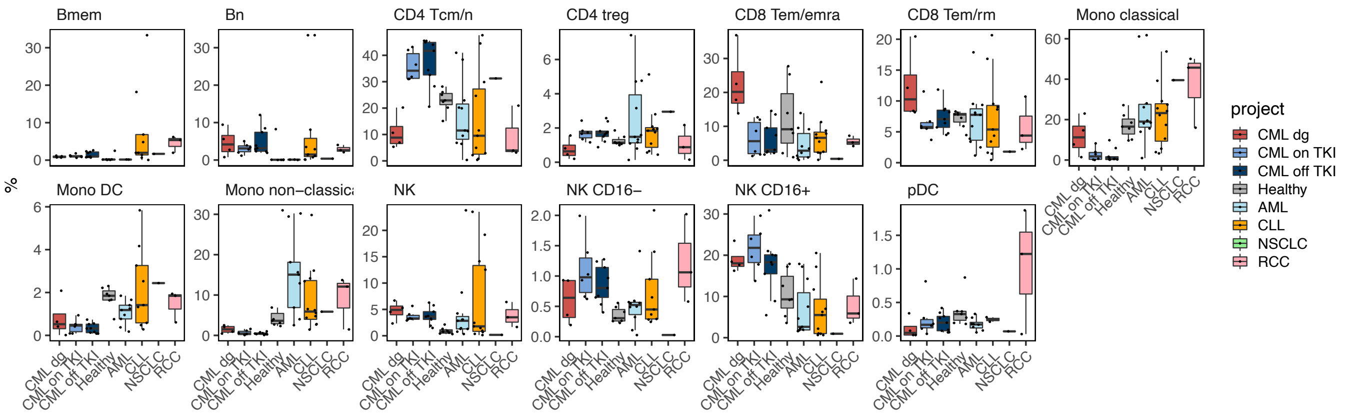

b

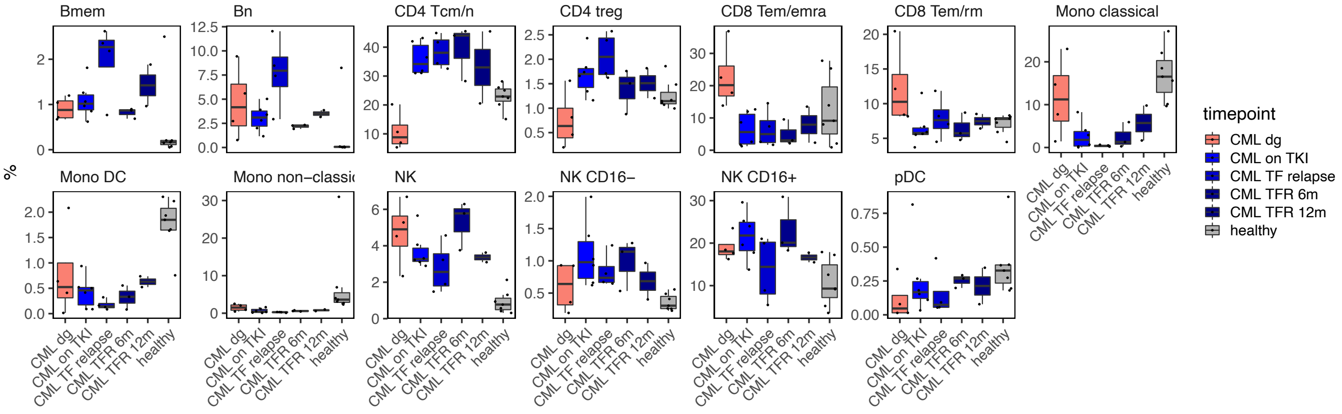

Supplementary Figure 4: ScRNAseq population abundances in patients with CML in different phases and healthy

a) ScRNAseq population abundances in patients with CML (diagnosis *n*=4, on TKI *n*=6, *N*=6, off TKI *n*=6, *N*=10), healthy controls (*n*=7), patients with untreated hematological cancers (CLL *n*=13, AML *n*=11), and patients with untreated solid cancer (RCC *n*=3, NSCLC *n*=1).

b) ScRNAseq population abundances in patients with CML (diagnosis *n*=4, on TKI *n*=6, *N*=6, off TKI *n*=6, *N*=10) and healthy controls (*n*=7). In off TKI samples, patients were either in treatment-free remission (TFR, *n*=2) or encountered a treatment-free (TF) relapse (*n*=4), which happened either early (< 6 months) or late (> 6 months) following the TKI cessation. *P*-values were not calculated due to low number of samples.

CML=chronic myeloid leukemia, CLL=chronic lymphocytic leukemia, AML= acute myeloid leukemia, RCC=renal cell carcinoma, NSCLC=non-small cell lung carcinoma, TKI=tyrosine kinase inhibitor. *n* refers to the number of patients and *N* to the number of samples where it differs from *n*.

Supplementary Figure 5

a

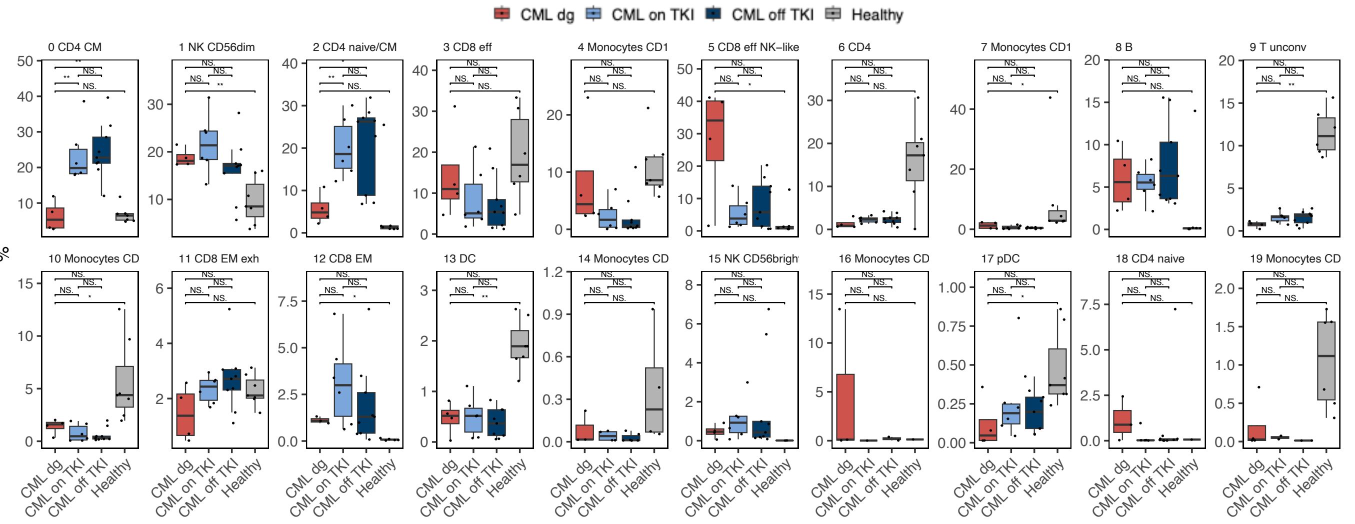

b

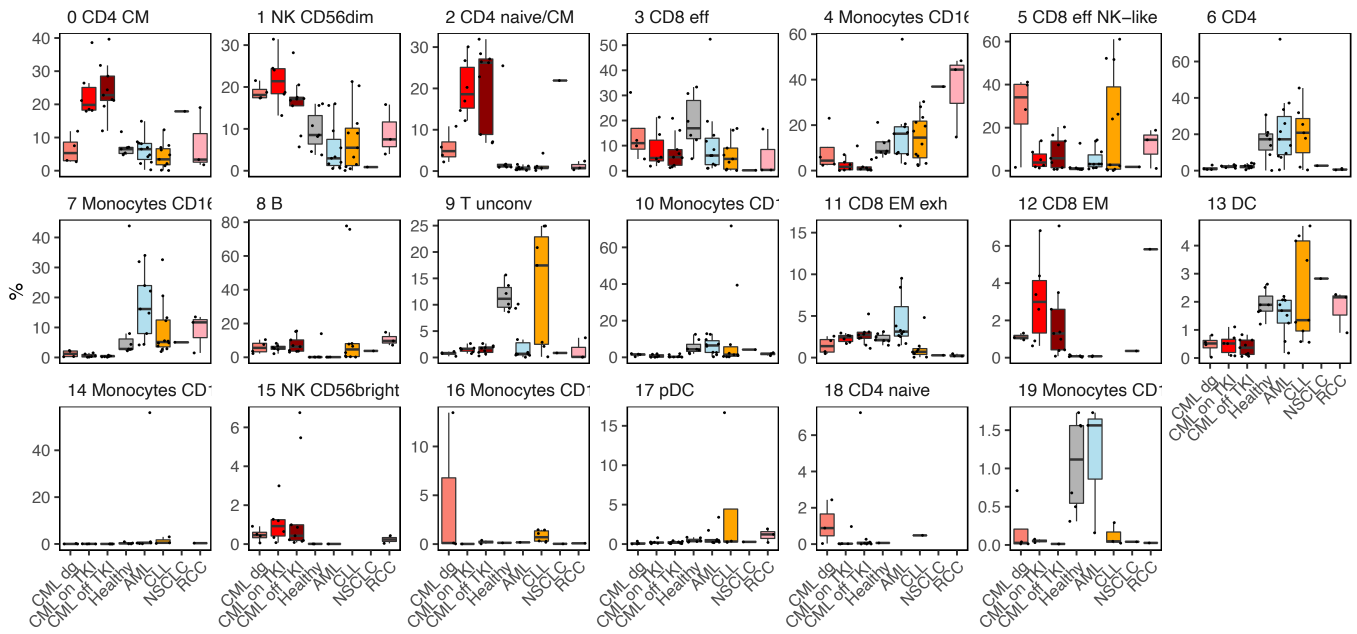

Supplementary Figure 5: ScRNAseq population abundances in patients with CML, healthy, and patients with different cancers with cluster-based solution

a) ScRNAseq cluster abundances in patients with CML (diagnosis *n*=4, on TKI *n*=6, *N*=6, off TKI *n*=6, *N*=10) and healthy controls (*n*=7). *P*-values were calculated with two-sided Mann-Whitney test.

b) ScRNAseq cluster abundances in patients with CML (diagnosis *n*=4, on TKI *n*=6, *N*=6, off TKI *n*=6, *N*=10), healthy controls (*n*=7), patients with untreated hematological cancers (CLL *n*=13, AML *n*=11), and patients with untreated solid cancer (RCC *n*=3, NSCLC *n*=1). *P*-values were calculated with Kruskal-Wallis test. CML=chronic myeloid leukemia, CLL=chronic lymphocytic leukemia, AML= acute myeloid leukemia, RCC=renal cell carcinoma, NSCLC=non-small cell lung carcinoma, TKI=tyrosine kinase inhibitor. *n* refers to the number of patients and *N* to the number of samples where it differs from *n*.

Supplementary Figure 6

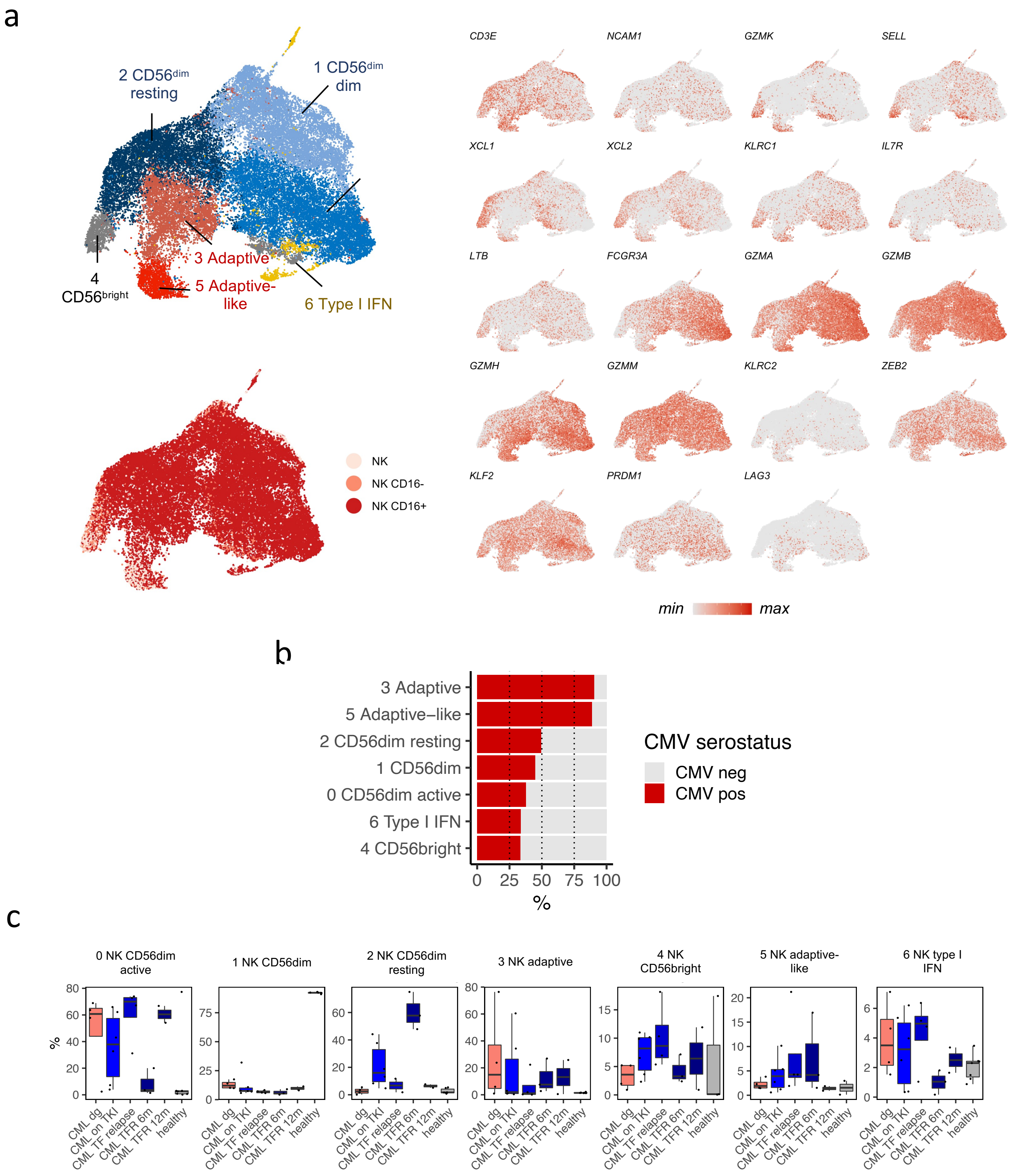

Supplementary Figure 6: Detailed NK cell clusters

a) UMAP representation of the NK CD16+, NK CD16- and NK cells identified by Celltypist, colored by manually annotated clusters (top), Celltypist predicted celltypes (bottom) or scaled expression of genes used to annotate the phenotypes (right).

b) Proportion of cells from CMV seropositive (*n*=4) and seronegative (*n*=2) patients with CML. The number of cells were subsampled to be the same between the two patient groups.

c) ScRNAseq population abundances in patients with CML (diagnosis *n*=4, on TKI *n*=6, *N*=6, off TKI *n*=6, *N*=10) and healthy controls (*n*=7). In off TKI samples, patients were either in treatment-free remission (TFR, *n*=2) or encountered a treatment-free (TF) relapse (*n*=4), which happened either early (< 6 months) or late (> 6 months) following the TKI cessation. *P*-values were not calculated due to low number of samples.

Supplementary Figure 7

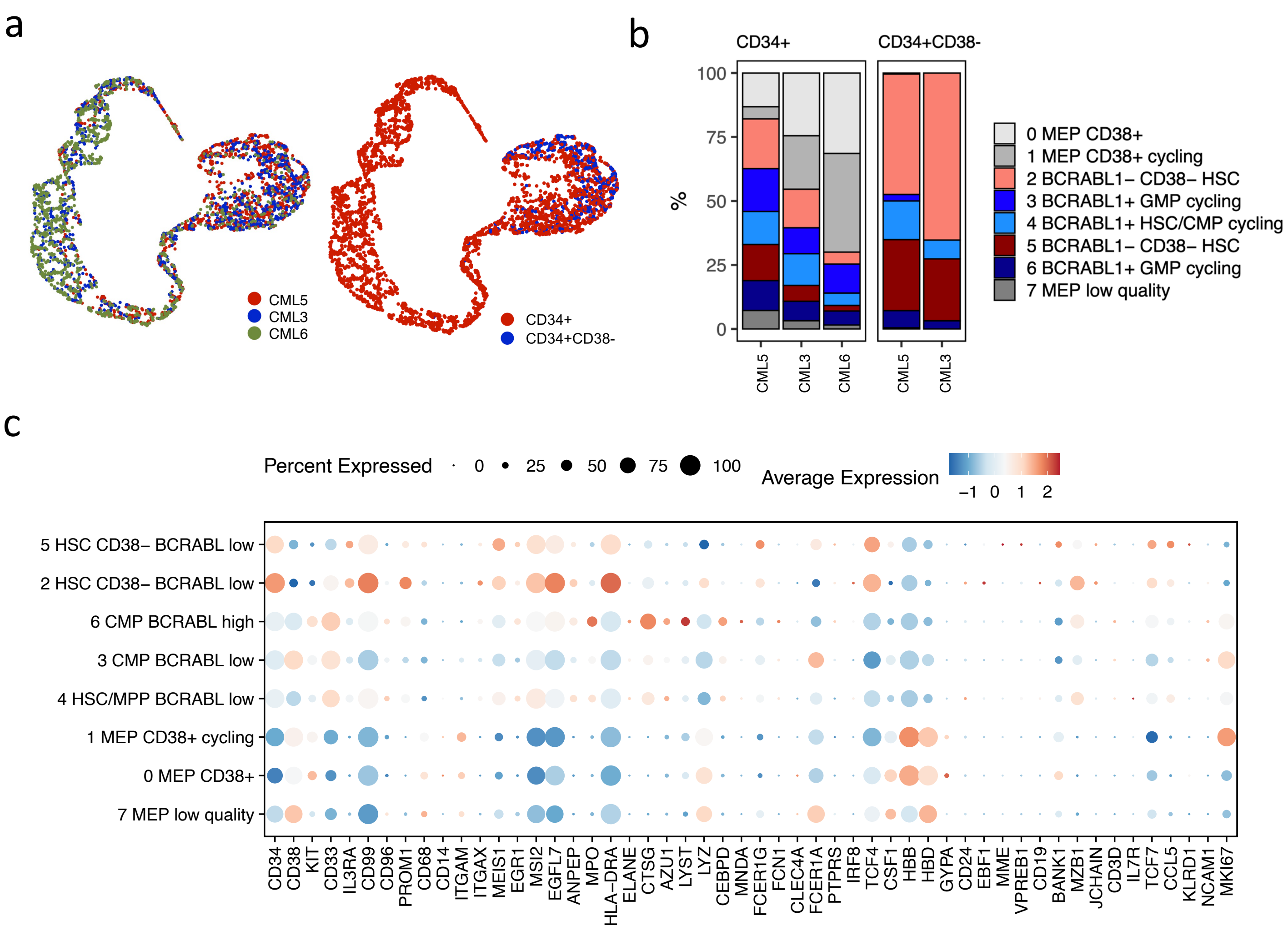

Supplementary Figure 7: Annotation of CD34+ and CD34+CD38- single-cell RNA-sequencing samples

a) UMAP representation of the transcriptomic profile of pooled CD34+ (*n*=3) and CD34+CD38- sorted samples (*n*=2), colored by patient or sorting strategy.

b) Proportion of cells from CD34+ and CD34+CD38- sorted samples.

c) Scaled average expressions and proportion of cells expressing the canonical markers used to aid to define the clusters, adopted from Van Galen et al publication[10].

Supplementary Figure 8

a

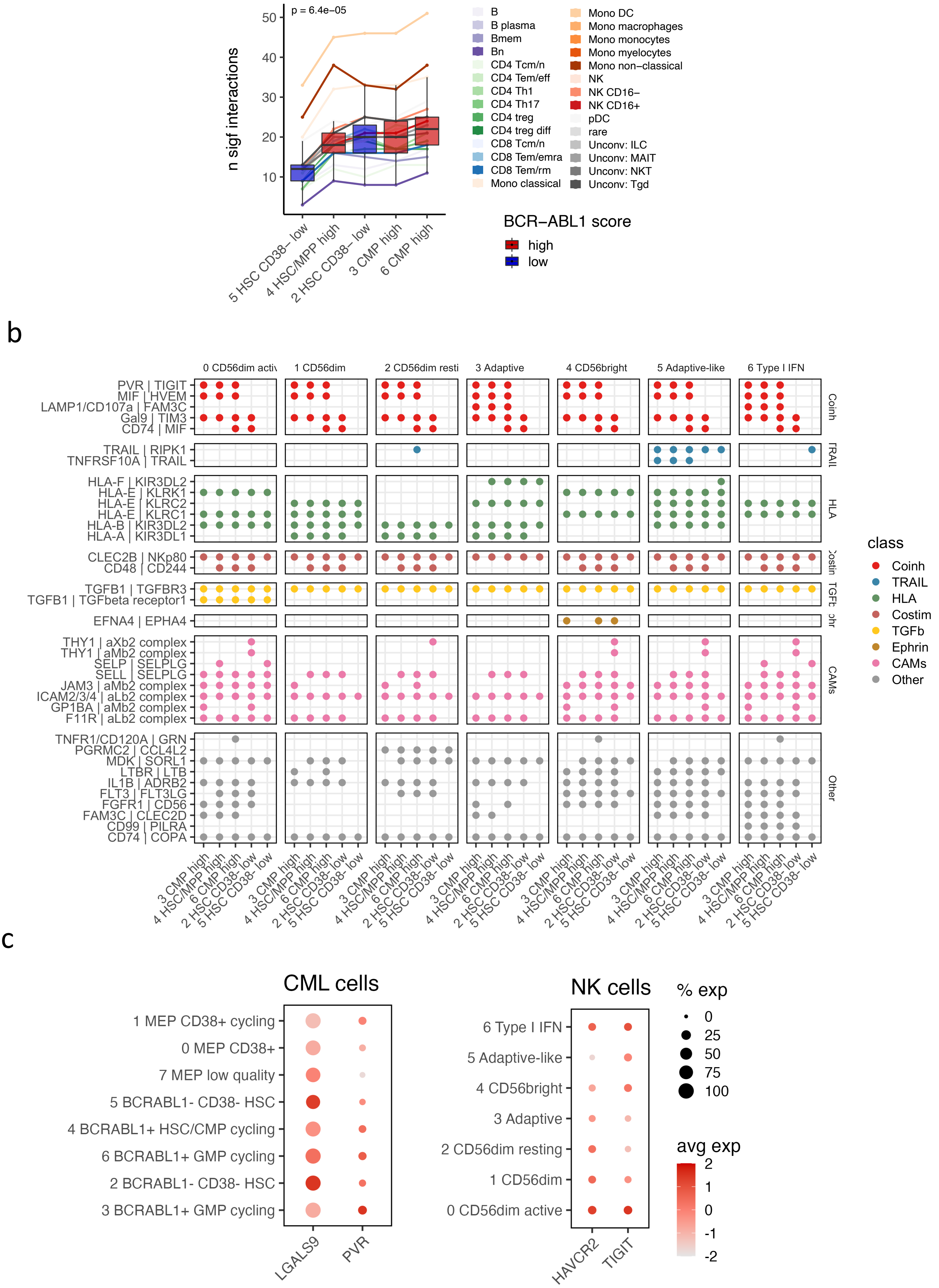

Supplementary Figure 8: Immune cell – cancer cell interactions

a) Number of significant (*P*<0.05, CellPhoneDB test) ligand-receptor interactions between CML cells and immune cells from patients with newly diagnosed CML. In the box plot, each dot is one immune cell type. *P*-value calculated with Kruskal-Wallis test.

b) All significant (*P*<0.05, CellPhoneDB test) ligand-receptor interactions between CML and NK cells.

c) Scaled average expressions and proportion of cells expressing *LGALS9* – *HAVCR2*/TIM3 and *PVR* – *TIGIT.*

Supplementary Figure 9

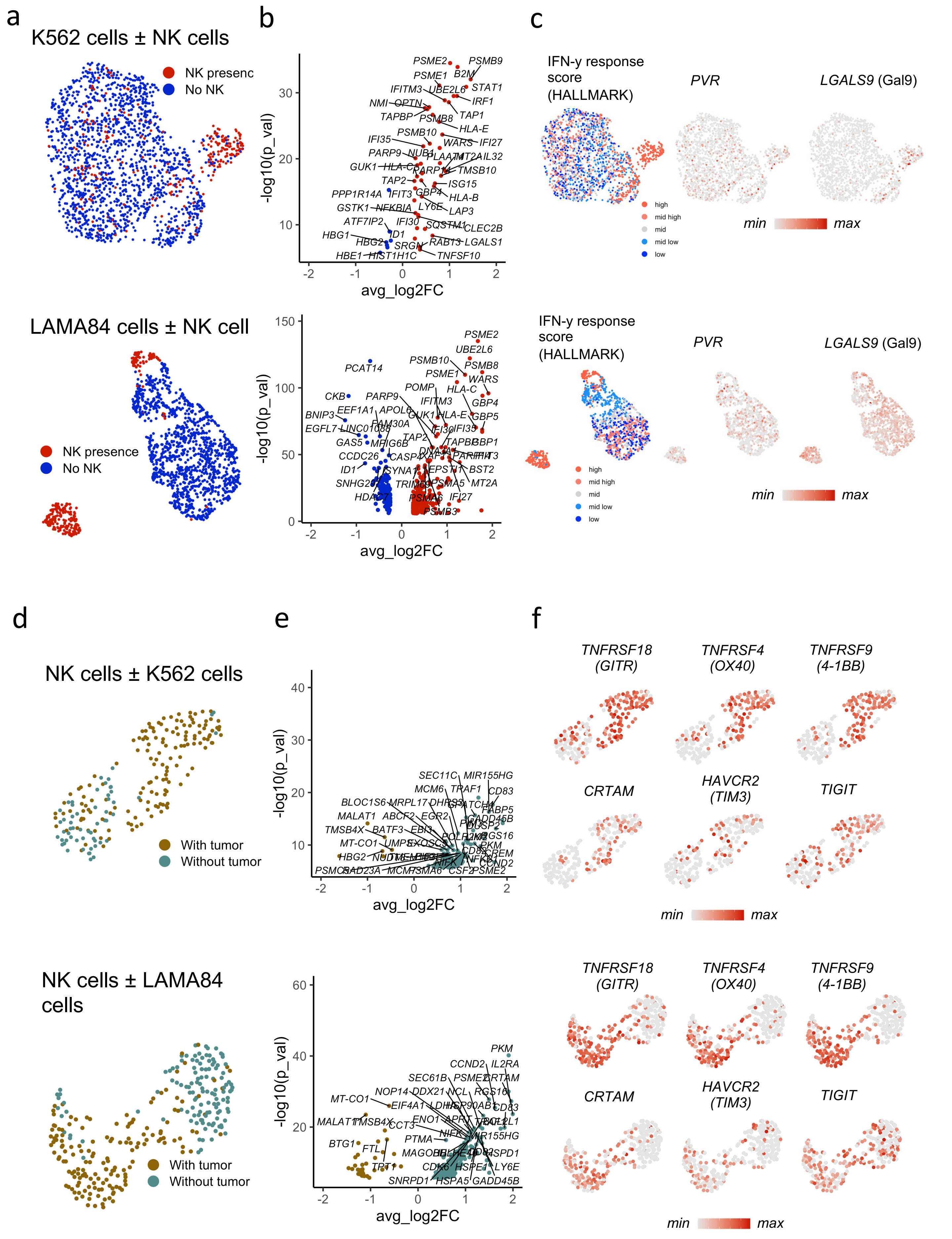

Supplementary Figure 9: Co-culture of primary NK cells with CML cell lines

a) UMAP representation of K562 and LAMA84 cells cultured with and without non-expanded NK cells, colored by the presence of expanded NK cells.

b) Differentially expressed genes (P_{adj}<0.05, Bonferroni corrected *t*-test) between K562 and LAMA84 cells cultured with and without non-expanded NK cells,

c) Expression of selected co-inhibitory genes and IFN-y response score in K562 and LAMA84 cells, calculated with the HALLMARK-category genes.

d) UMAP representation of non-expanded NK cells cultured with and without K562 or LAMA84 cells, colored by the presence of tumor cells.

e) Differentially expressed genes (P_{adj}<0.05, Bonferroni corrected *t*-test) between non-expanded NK cells cultured with and without K562 or LAMA84 cells.

f) Expression of selected co-stimulatory genes in non-expanded NK cells.

Supplementary Figure 10

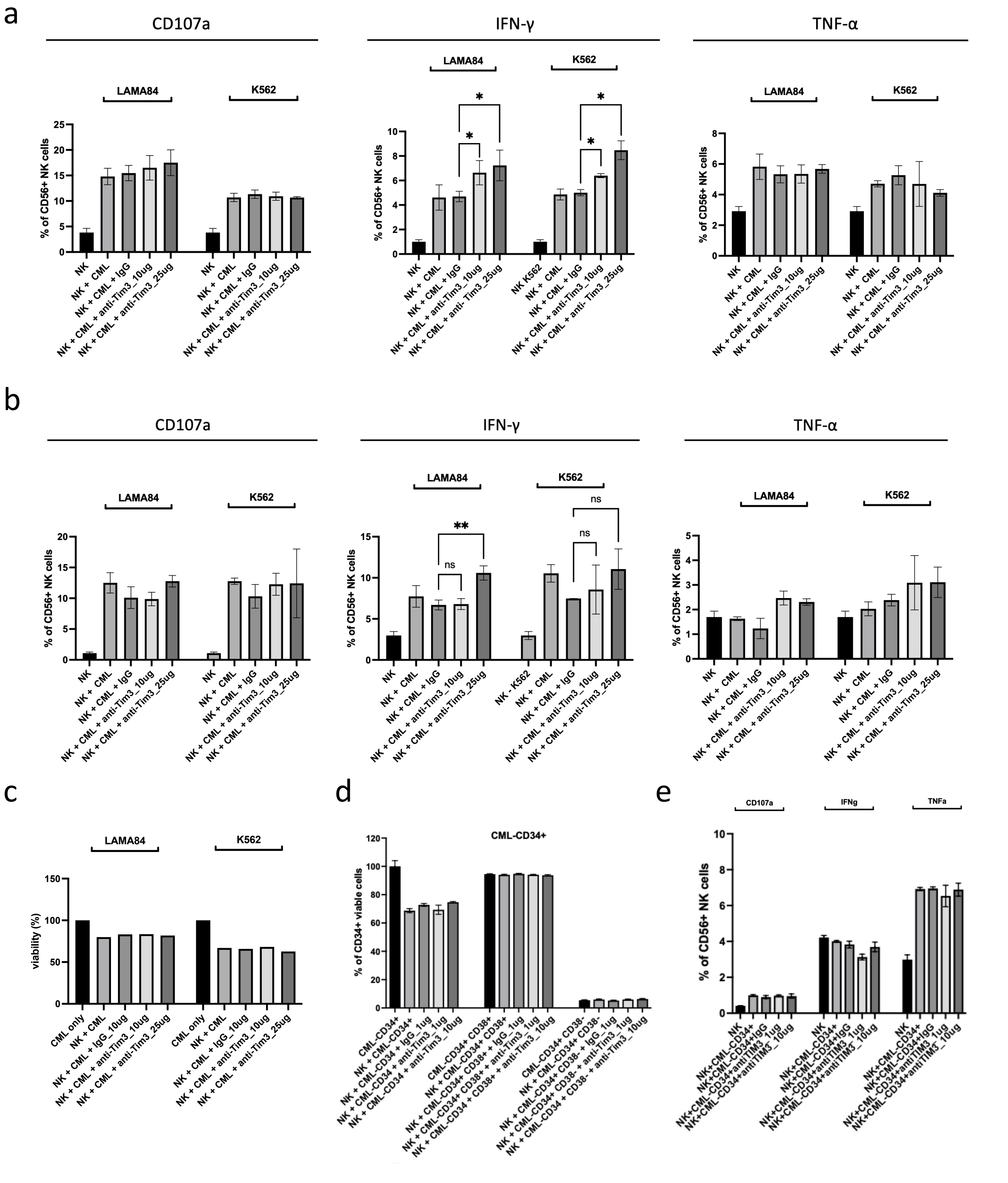

Supplementary Figure 10: Blocking TIM3 in CML cell lines

a) Proportion of expanded NK cells from healthy donor expressing CD107a (left), IFN-γ (middle), or TNF-α (right) after co-culture with CML cell lines LAMA84 or K562 with or without TIM3 antibody in different concentrations. *P*-values were calculated with a paired *t*-test.

b) Proportion of expanded NK cells from another healthy donor expressing CD107a (left), IFN-γ (middle), or TNF-α (right) after co-culture with CML cell lines LAMA84 or K562 with or without TIM3 antibody in different concentrations. *P*-values were calculated with a paired *t*-test.

c) Proportion of viable CML cells from K562 or LAMA84 cell lines after co-culture with expanded NK cells from panel B with or without TIM3 antibody in different concentrations.

d) Proportion of viable primary CML cells expressing CD34+ (left), CD34+CD38+ (middle), or CD34+CD38- (right) co-cultured with expanded NK cells from panel B with or without TIM3 antibody in different concentrations.

e) Proportion of expanded NK cells from healthy donor expressing CD107a (left), IFN-γ (middle), or TNF-α (right) after co-culture with primary CML cells from panel D with or without TIM3 antibody in different concentrations.

*=P<0.05, **=P<0.01, ***=P<0.001

Supplementary Figure 11

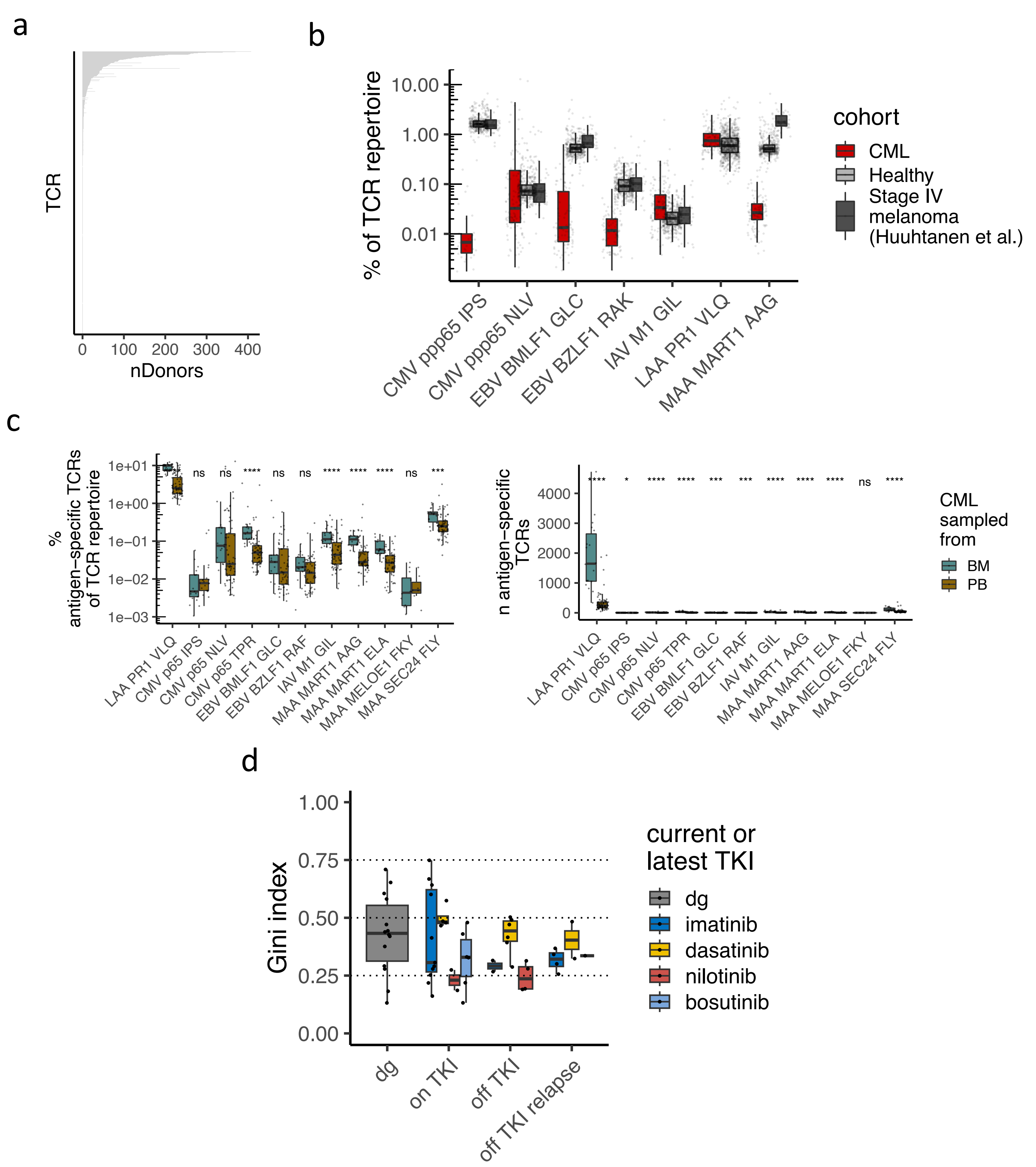

Supplementary Figure 11: Proportion and number of antigen-specific TCRs

a) Bar plot showing the number of healthy donors (*n*=783) harboring a given PR1-specific TCR.

b) Percentages of TCR repertoire containing TCRGP predicted antigen-specific TCRs between patients with CML (*n*=48), healthy donors (*n*=786) from Emerson et al. and patients with stage IV melanoma (*n*=46), from Huuhtanen et al.. All samples are from peripheral blood and subsampled to same read depth (40,000 reads).

c) The proportion and number of antigen-specific TCRs between peripheral blood (PB, *n*=24) and bone marrow (BM, *n*=15) samples from patients with CML.

d) TCR-repertoire clonality in patients with CML in diagnosis (*n*=14), on TKI (*n*=23), off TKI (*n*=12), off TKI relapse (*n*=7). High Gini coefficients denotes high clonality.

Supplementary Figure 12

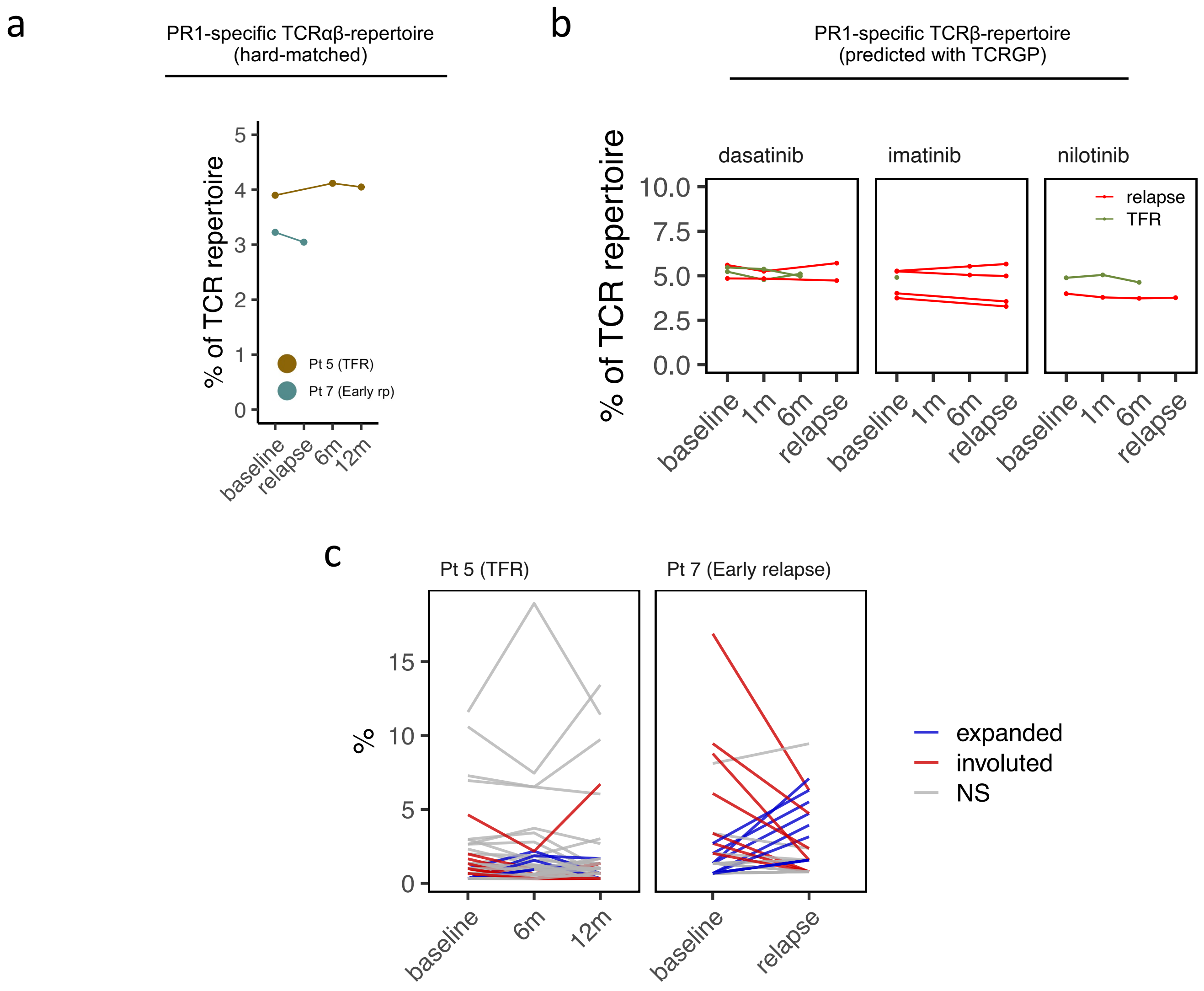

Supplementary Figure 12: Clonal dynamics of anti-PR1 T cells in patients with CML

a) Proportion of hard-matched anti-PR1 TCRs in CML patients after TKI cessation profiled with scRNA+TCRαβ-seq (*n*=2). Patients were treated with imatinib before TKI cessation.

b) Proportion of TCRGP predicted anti-PR1 TCRs in CML patients after TKI cessation profiled with TCRβ-seq (*n*=11). Patients were treated with different TKIs (dasatinib, imatinib, nilotinib) before TKI cessation.

c) Proportion of hard-matched anti-PR1 TCRs in clones in patients after TKI cessation profiled with scRNA+TCRαβ-seq (*n*=2). Percentages are out of anti-PR1 TCR repertoire. Patients were treated with imatinib before TKI cessation.
